# Mutation of *daf*‐*2* extends lifespan via tissue‐specific effectors that suppress distinct life‐limiting pathologies

**DOI:** 10.1111/acel.13324

**Published:** 2021-02-20

**Authors:** Yuan Zhao, Bruce Zhang, Ioan Marcu, Faria Athar, Hongyuan Wang, Evgeniy R. Galimov, Hannah Chapman, David Gems

**Affiliations:** ^1^ Research Department of Genetics, Evolution and Environment Institute of Healthy Ageing University College London London UK

**Keywords:** aging, *Caenorhabditiselegans*, genetics, insulin/IGF‐1 signaling

## Abstract

In aging *Caenorhabditis elegans*, as in higher organisms, there is more than one cause of death. *C*. *elegans* exhibit early death with a swollen, infected pharynx (P death), and later death with pharyngeal atrophy (p death). Interventions that alter lifespan can differentially affect frequency and timing of each type of death, generating complex survival curve shapes. Here, we use mortality deconvolution analysis to investigate how reduction of insulin/IGF‐1 signaling (IIS), which increases lifespan (the Age phenotype), affects different forms of death. All *daf*‐*2* insulin/IGF‐1 receptor mutants exhibit increased lifespan in the p subpopulation (p Age), while pleiotropic class 2 *daf*‐*2* mutants show an additional marked reduction in P death frequency. The latter is promoted by pharyngeal expression of the IIS‐regulated DAF‐16 FOXO transcription factor, and at higher temperature by reduced pharyngeal pumping rate. Pharyngeal DAF‐16 also promotes p Age in class 2 *daf*‐*2* mutants, revealing a previously unknown role for the pharynx in the regulation of aging. Necropsy analysis of *daf*‐*2* interactions with the *daf*‐*12* steroid receptor implies that previously described opposing effects of *daf*‐*12* on *daf*‐*2* longevity are attributable to internal hatching of larvae, rather than complex interactions between insulin/IGF‐1 and steroid signaling. These findings support the view that wild‐type IIS acts through multiple distinct mechanisms which promote different life‐limiting pathologies, each of which contribute to late‐life mortality. This study further demonstrates the utility of mortality deconvolution analysis to better understand the genetics of lifespan.

## Graphical abstract

Different *daf‐2* insulin/IGF‐1 receptor mutations differentially affect different forms of death in *C. elegans.* This involves tissue‐ and isoform‐specific action of the DAF‐16 FOXO transcription factor, notably in the pharynx where it protects not only against lethal pharyngeal pathology, but also aging more widely. Thus, wild‐type *daf‐2* promotes death from old age through multiple effectors with distinct effects on the various senescent pathologies that cause it.

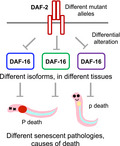

## 1 INTRODUCTION

**Figure 1:**
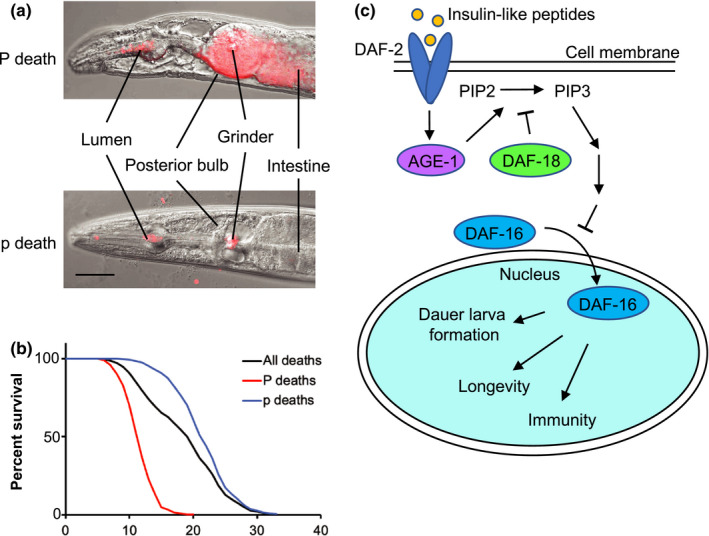
Schematic of mortality deconvolution and insulin/IGF‐1 signaling (IIS) pathway in *Caenorhabditis elegans*. (a) Representative Nomarski images of P and p corpses of worms fed RFP‐expressing *E. coli*. P corpses are characterized by the widespread RFP signal in the pharyngeal tissue, while p corpses contain either limited RFP foci or only signal in the grinder lumen. Scale bar 40 μm. (b) Schematic of mortality deconvolution for a typical wild‐type *C. elegans* lifespan assay. The black survival curve represents survival of all animals in the population, red and blue curves represent that of the P and p death subpopulation, respectively. (c) Simplified schematic of the IIS pathway in *C. elegans*. Under optimal conditions, insulin‐like peptides activate the DAF‐2 insulin/IGF‐1 receptor and its downstream kinase AGE‐1/PI3 K, which converts PIP2 to PIP3, an activity antagonized by DAF‐18/PTEN. Through a cascade of phosphorylation increased PIP3 level leads to phosphorylation and cytosolic sequestration of DAF‐16/FOXO. When IIS is inhibited, DAF‐16 enters the nucleus where it alters expression of genes involved in dauer formation, longevity, and innate immunity

Many genes have been identified manipulation of which can alter lifespan in the nematode *Caenorhabditis elegans*. By comparison, the proximate mechanisms that such genes influence to alter lifespan remain poorly understood. Lifespan is a function of senescent pathology that causes death (Blagosklonny, 2007; Gems, 2015). In *C. elegans*, a subset of aging wild‐type hermaphrodites die as the result of a lethal pharyngeal infection (Zhao et al., 2017). This is detectable by means of necropsy after death from old age, as corpses with an enlarged, swollen pharynx (P death ["big P"]). Worms that escape P death die later with an atrophied pharynx (p death ["small p"]) (Figure 1a). Genetic mutations or treatments that change lifespan act by differentially altering the frequency and/or timing of each form of death. By combining mortality and necropsy analysis, effects of interventions on P and p subpopulations can be assessed (mortality deconvolution) (Zhao et al., 2017) (Figure 1b).

The evolutionarily conserved IIS pathway affects a variety of biological processes, including development and aging (Figure 1c). In *C. elegans*, mutation of the *daf*‐*2* insulin/IGF‐1 receptor or the *age*‐*1* phosphatidylinositol 3‐kinase (PI3 K) catalytic subunit can cause constitutive formation of diapausal dauer larvae (the Daf‐c phenotype) (Murphy & Hu, 2013) and greatly extended lifespan (the Age phenotype) (Friedman & Johnson, 1988; Kenyon et al., 1993). Reduced IIS can also cause proportionally more modest increases in lifespan in higher organisms, including fruit flies, mice, and possibly humans (Kenyon, 2010). While the mechanisms mediating effects of IIS on lifespan remain uncertain, reduced IIS must act by altering the frequency and/or timing of P and/or p death. A further possibility is that effects of IIS may be mediated by mechanisms specific to either the P or p subpopulation.

Genetic analysis of *daf*‐*2* is complicated by complex differences between *daf*‐*2* alleles, which can be broadly grouped into two classes. Class 1 mutants have lesions in extracellular regions of the DAF‐2 receptor, and Age is partially suppressed by mutation of the *daf*‐*12* nuclear hormone receptor. By contrast, class 2 mutants have lesions in either the DAF‐2 receptor ligand‐binding domain or the intracellular tyrosine kinase domain, are more pleiotropic, showing temperature‐sensitive reductions in feeding rate, movement rate and fertility, and the Age phenotype is often increased by mutation of *daf*‐*12* (Antebi et al., 2000; Gems et al., 1998; Larsen et al., 1995; Patel et al., 2008).

Both the Daf‐c and Age phenotypes of IIS mutants require the DAF‐16/FOXO transcription factor (Kenyon et al., 1993; Murphy & Hu, 2013). In well‐nourished worms, DAF‐16 is phosphorylated and sequestered in the cytosol, but moves into the nucleus under stressed conditions and when IIS is reduced (Murphy & Hu, 2013), retarding aging (Figure 1c). The genetics of *daf*‐*16* is complicated by the presence of multiple isoforms. Three sets of *daf*‐*16* isoforms are transcribed from distinct promoters in distinct tissues, *daf*‐*16a*, *daf*‐*16b*, and *daf*‐*16d*/*f*/*h* (henceforth referred to as *daf*‐*16f*) (Bansal et al., 2014; Kwon et al., 2010; Murphy & Hu, 2013). Of these *daf*‐*16a* is the main mediator of *daf*‐*2* Age, Daf‐c, and stress resistance, with *daf*‐*16f* also playing a lesser role; in tests with *daf*‐*16* isoform‐specific mutations, *daf*‐*16a*(‐) but not *daf*‐*16f*(‐) partially suppressed *daf*‐*2* Daf‐c and Age, but simultaneous abrogation of both isoforms strongly suppressed both phenotypes (Chen et al., 2015). Earlier evidence for a predominant role of *daf*‐*16f* (Bansal et al., 2014; Kwon et al., 2010) seems to have been an artifact of *daf*‐*16f* transgene overexpression and failure of the *daf*‐*16a* transgene to fully recapitulate *daf*‐*16a* isoform function (Chen et al., 2015). *daf*‐*16b* plays only a minor role in dauer development (Lee et al., 2001; Lin et al., 2001). Earlier studies focusing on *daf*‐*16a* demonstrated the importance of intestinal and neuronal DAF‐16 expression in *daf*‐*2* longevity, but also suggested that the full extent of *daf*‐*2* Age requires DAF‐16 function in other, unidentified tissues (Libina et al., 2003).

Here, we use mortality deconvolution analysis to reveal distinct effects of IIS on aging in P and p subpopulations, shedding new light on earlier lifespan genetic studies. These results reveal how distinct elements of IIS act differentially and in condition‐dependent ways upon distinct mechanisms that contribute to illness and death in elderly worms.

## 2 RESULTS

### 2.1 Reduced *daf*‐*2* function reduces P death and causes p Age in an allele‐ and temperature‐dependent fashion

To investigate how IIS affects the two forms of death in *C. elegans*, we performed survival, necropsy, and mortality deconvolution analysis on a range of *daf*‐*2* loss‐of‐function mutants: three class 1 (*m41*, *m577*, and *e1368*), two class 2 (*e1370* and *m120*), and *m596* which is at the boundary between class 1 and class 2 (Gems et al., 1998; Patel et al., 2008). Trials were conducted at 15˚C and 20˚C, which are permissive for development, and at 25˚C which induces dauer entry during development and dauer‐like behavior in class 2 *daf*‐*2* mutant adults. All alleles were crossed into a *fln*‐*2(*+*)* background to avoid the confounding effects of the *fln*‐*2(ot611)* mutation, which reduces P frequency (Zhao et al., 2019). The resulting, deconvolved data includes survival data for the whole population, and for P and p subpopulations, plus the proportion of P deaths. However, because P sample sizes are often small, we discount P lifespan data in most cases, but present the data in Appendix S1. Raw survival data for all trials are available in Data S1.

**Figure 2:**
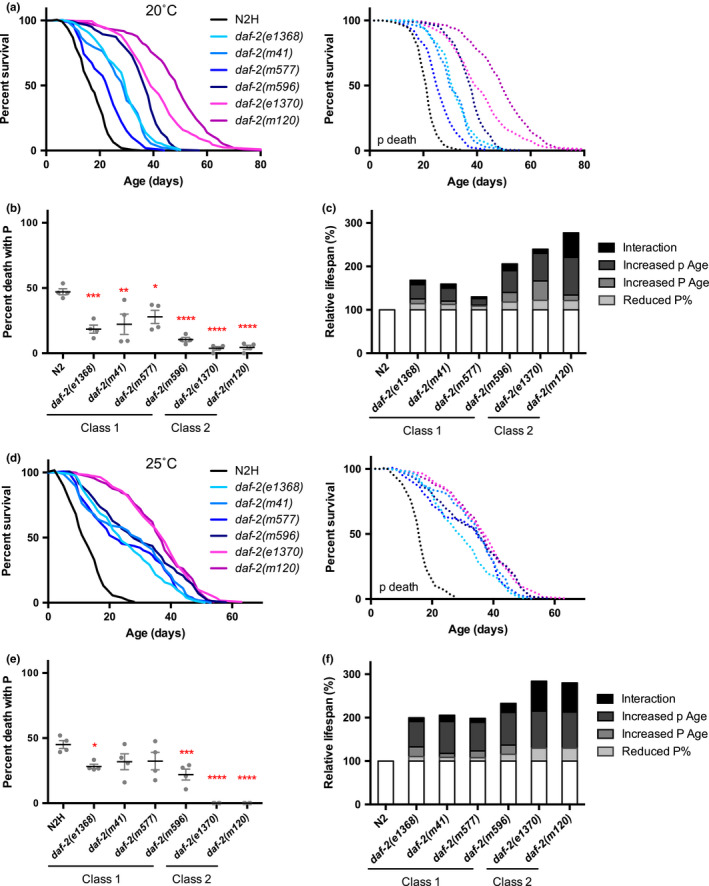
Effects of class 1 and 2 *daf*‐*2* alleles on lifespan and P frequency at 20˚C and 25˚C. (a) Lifespan of the whole population or the p subpopulation and (b) frequency of P death of class 1 and class 2 *daf*‐*2* mutants at 20˚C. *N* = 2 trials. (c) Contribution of increased p and P lifespan, and reduced P frequency, to *daf*‐*2* mutants lifespan at 20˚C. The contribution of each factor is calculated one‐variable‐at‐a‐time, and interaction represents the residue effect due to the non‐linear nature of lifespan. (d) Lifespan and (e) frequency of P death of class 1 and class 2 *daf*‐*2* mutants at 25˚C. *N* = 2 trials. (f) Contribution of increased p and P lifespan, and reduced P frequency, to *daf*‐*2* mutants lifespan at 25˚C

At 20˚C, all six *daf*‐*2* mutants showed an increase in overall lifespan whose magnitude varied both among and between the two classes (Figure 2a), similar to previously reported overall severity ranking of *daf*‐*2* alleles (Gems et al., 1998). All mutants showed p Age and reduced P frequency, both of which were more marked in class 2 mutants (Figure 2a,b; Appendix S1: Table S1). A similar result was observed at 15˚C, where both *e1368* and *e1370* significantly reduced P frequency and increased p lifespan (Appendix S1: Figure S1; Table S2). The relative contribution of reduced P frequency and increased P and p lifespan to the overall Age phenotype was also calculated for each mutant. This showed that the greater lifespan of class 2 alleles was largely attributable to greater p Age (Figure 2c).

At 25˚C, overall lifespan varied significantly between the two classes but, in contrast to 20˚C, was similar within each class of mutants (Figure 2d; Appendix S1: Table S3). Class 1 mutants again showed modest reduction in P frequency, but in class 2 mutants P death was entirely absent (Figure 2e). Moreover, the difference in p Age between the two classes is much smaller at 25˚C compared with 20˚C (Figure 2d,f; Appendix S1: Figure S2; Table S4). Thus, the greater longevity of *daf*‐*2(e1370)* and *daf*‐*2(m120)* is largely attributable to a reduction in the proportion of P deaths. Overall, these results show that the relative contribution of reduced P and of p Age to overall Age varies greatly between *daf*‐*2* mutants depending on allele and ambient temperature.

**Figure 3:**
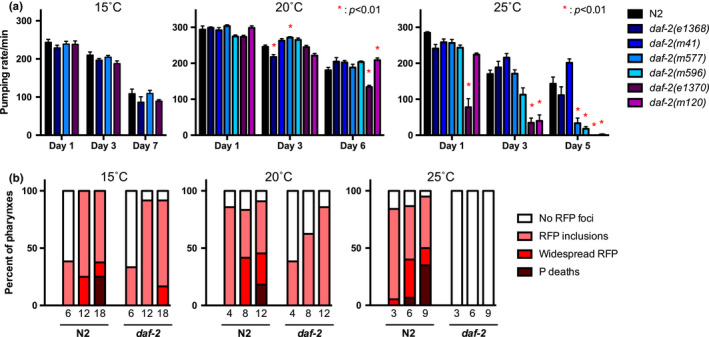
Temperature sensitivity of resistance to initial bacterial invasion and Eat in *daf*‐*2(e1370)*. These results are consistent with the possibility that class 2 *daf*‐*2* Eat contributes to resistance to P death. (a) Effects of mutation of *daf*‐*2* on pharyngeal pumping rates in early adulthood at 15˚C, 20˚C, and 25˚C. A reduction in pumping rate was consistently observed in class 2 mutants at 25˚C only, consistent with earlier findings (Gems et al., 1998; Kenyon et al., 1993). *n* ≥ 10. Star indicates *p* < 0.01. (b) *daf*‐*2(e1370)* had little effect on initial bacterial invasion but suppressed widespread infection at 15˚C, delayed both bacterial invasion and further infection at 20˚C, and fully suppressed bacteria invasion at 25˚C. *N* = 2 trials

Knockdown of *daf*‐*2* function by RNA‐mediated interference (RNAi) also increases *C. elegans* lifespan (Arantes‐Oliveira et al., 2003). *daf*‐*2* RNAi of wild‐type animals from either L4 or from the parental generation onward resulted in p Age but little reduction in P frequency (Appendix S1: Figure S3A, B). Similar results were obtained using the enhanced RNAi mutant *rrf*‐*3(pk1426)* (Simmer et al., 2002) (Appendix S1: Figure S3C, D). Differences between effects of *daf*‐*2* mutant alleles and *daf*‐*2* RNAi have been noted previously. For example, *daf*‐*2* RNAi fails to induce dauer entry at 25˚C, and *daf*‐*2* class 1 mutants and *daf*‐*2* RNAi show additive effects on lifespan (Arantes‐Oliveira et al., 2003; Ewald et al., 2015). The lack of an effect of *daf*‐*2* RNAi on P death could reflect non‐induction of the dauer program; however, reductions of pharyngeal infection and P death seen in *daf*‐*2(e1370)* at 15˚C (Figure 3b; Appendix S1: Figure S1) suggest that some suppression of P death can occur in the absence of the dauer program. The distinct effects of *daf*‐*2* RNAi could reflect a difference in the way that IIS is disrupted within cells (see Discussion) and/or different levels of disruption in different tissues.

### 2.2 *daf*‐*2* can reduce P death independently of effects on feeding rate

At 22.5˚C and 25˚C, class 2 *daf*‐*2* mutants exhibit a near cessation of feeding (pharyngeal pumping) in class 2 mutants (Gems et al., 1998). Moreover, *eat*‐*2* mutants exhibit reduced pharyngeal pumping rate and also reduced P frequency, apparently due to reduced mechanical damage to the pharyngeal cuticle (Zhao et al., 2017), an example of mechanical senescence. This suggests that the different levels of suppression of P death in *daf*‐*2* mutants could reflect different degrees of suppression of pharyngeal pumping. To explore this, we assayed the effect of *daf*‐*2* on pumping rate. At 25˚C, pumping rate in class 2 mutants was greatly reduced by day 3 of adulthood, but little reduction in pumping was seen at 15˚C or 20˚C, broadly consistent with previous findings (Gems et al., 1998). Thus, the reductions in P frequency seen in *daf*‐*2* mutants at 15˚C and 20˚C are not attributable to reduced pharyngeal pumping rate.

During early adulthood, low‐level infection of pharyngeal tissue occurs, apparently due to activity‐dependent mechanical damage to the pharyngeal cuticle. This early infection leads in later life to the gross infection that causes P death and can be visualized using RFP‐expressing *E. coli* OP50 as red fluorescent inclusions or puncta within pharyngeal tissue (Zhao et al., 2017) (Figure 1a). We asked: does *daf*‐*2(e1370)* prevent early pharyngeal infection? As expected, at 25˚C initial bacterial invasion was fully suppressed, while at 20˚C *daf*‐*2(e1370)* caused a marked reduction in RFP inclusions in early adulthood (Figure 3b). Notably at 15˚C, early invasion was not suppressed but the progression to widespread infection is delayed and reduced (Figure 3b). Taken together these results suggest that *daf*‐*2(e1370)* acts in two way to reduce P death: by suppressing pumping, and also by a pumping rate‐independent mechanism, possibly improved innate immunity (see Discussion).

### 2.3 *daf*‐*2* gain‐of‐function increases P death frequency

**Figure 4:**
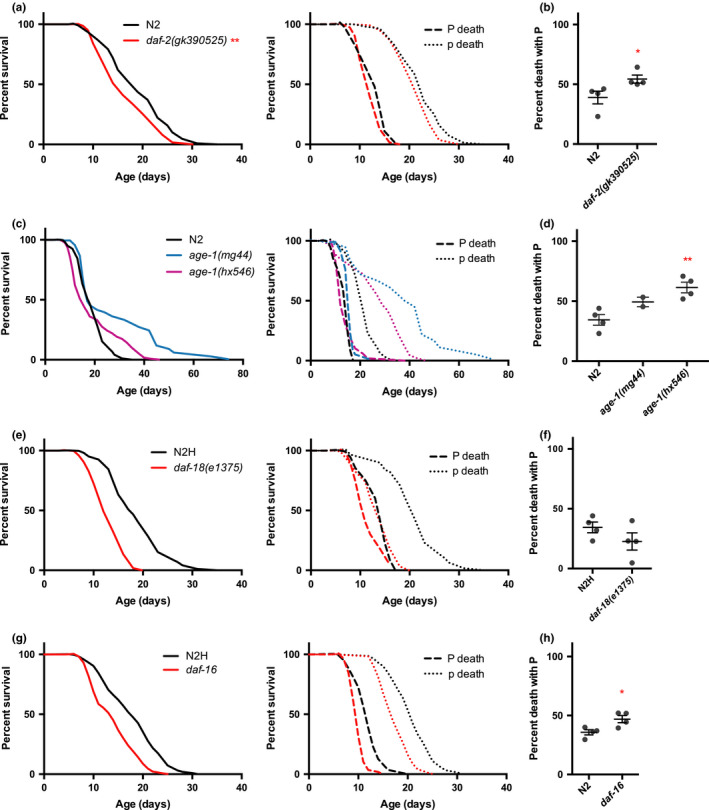
Effects of *daf*‐*2* gain‐of‐function mutant, *age*‐*1*, and *daf*‐*18* mutants on lifespan. (a) Lifespan and (b) P frequency of *daf*‐*2(gk390525)* gain‐of‐function mutant. *daf*‐*2(gf)* shortened lifespan largely by increasing P frequency. *N* = 2 trials. (c) Lifespan and (d) P frequency of two *age*‐*1* mutant alleles. *mg44* homozygotes were identified from among *age*‐*1* ^−/+^ mothers by singling worms and identifying those with all dauer progeny (see Methods). The increase in P frequency seen in the *age*‐*1(hx546)* strain was unexpected and could be an effect of *hx546* or possibly a difference in the genetic background. *N* = 3 trials. Regarding the relatively small effect of *hx546* on lifespan observed here: note that the effect of *hx546* on lifespan is temperature sensitive, showing larger effects at 25˚C than 20˚C (Dorman et al., 1995; Friedman & Johnson, 1988). Moreover, the classic Friedman and Johnson (1988) study employed monoxenic liquid culture with a lower *E. coli* concentration than plate culture, which may have reduced P death. (e) Lifespan and (f) P frequency of *daf*‐*18* mutant. *N* = 2 trials. (g) Lifespan and (h) P frequency of *daf*‐*16* null allele. *N* = 2 trials

The effects of mutation of *daf*‐*2* imply that IIS increases P death frequency and causes earlier p death. To test this further, we increased *daf*‐*2* activity level using the gain‐of‐function allele *daf*‐*2(gk390525gf)*, which increases DAF‐2 protein levels due to a defect in ubiquitylation‐mediated degradation and reduces lifespan (Tawo et al., 2017). As expected, this caused a modest but significant reduction in overall mean lifespan (−12%, *p* = 0.0028). Unexpectedly, this was largely due to a 40% increase in P frequency (*p* = 0.0486), while p lifespan was not significantly reduced (−4.8%, *p* = 0.103) (Figure 4a,b; Appendix S1: Figure S4A; Table S5). Consistent with this, when P death was suppressed using the antibiotic carbenicillin, p lifespan was not significantly reduced by *daf*‐*2(gf)* (−2.5%, *p* = 0.0906) (Appendix S1: Figure S4B). The increase in P frequency was not due to effects of *daf*‐*2* gain‐of‐function on pharyngeal pumping rate, since no increase was observed in the mutant (Appendix S1: Figure S4C). The implications of these findings are discussed in the next section.

### 2.4 *age*‐*1(rf)* and *daf*‐*18(rf)* increase and decrease p lifespan, respectively

The AGE‐1 PI3 K catalytic subunit acts downstream of DAF‐2 (Figure 1c), and mutation of *age*‐*1* increases lifespan (Friedman & Johnson, 1988; Morris et al., 1996). Mutation of *age*‐*1*, like that of *daf*‐*2*, caused p Age but did not reduce P frequency in either the *age*‐*1(hx546)* reduction‐of‐function mutant or the *age*‐*1(mg44)* null mutant (Figure 4c,d; Appendix S1: Table S6). The lack of a reduction in P death frequency is consistent with the neck‐and‐shoulder survival curve morphology seen in previous reports on *age*‐*1* (Friedman & Johnson, 1988; Tissenbaum and Ruvkun 1998), that is, with an initial steep decline in survival (due to P deaths) followed by sustained survival in the longer‐lived p subpopulation. Mutation of *glp*‐*1* also has this effect (Zhao et al., 2017).

Conversely, *daf*‐*18(e1375)*, a reduction‐of‐function mutation affecting the *C*. *elegans* PTEN PIP_3_ phosphatase that antagonizes the PI3 K pathway (Ogg & Ruvkun, 1998), shortened p lifespan but did not increase P frequency (Figure 4e,f; Appendix S1: Table S7). These results, taken together with the effects of *daf*‐*2(gf)*, could imply that p lifespan is a function of PIP_3_ levels, controlled by PI3 K and PTEN, while other outputs of the DAF‐2 receptor impact P frequency. Another possibility is that IIS increase by *daf*‐*2(gk390525)* is neomorphic in character.

### 2.5 Action of DAF‐16 in the pharynx reduces P death and promotes p Age

**Figure 5:**
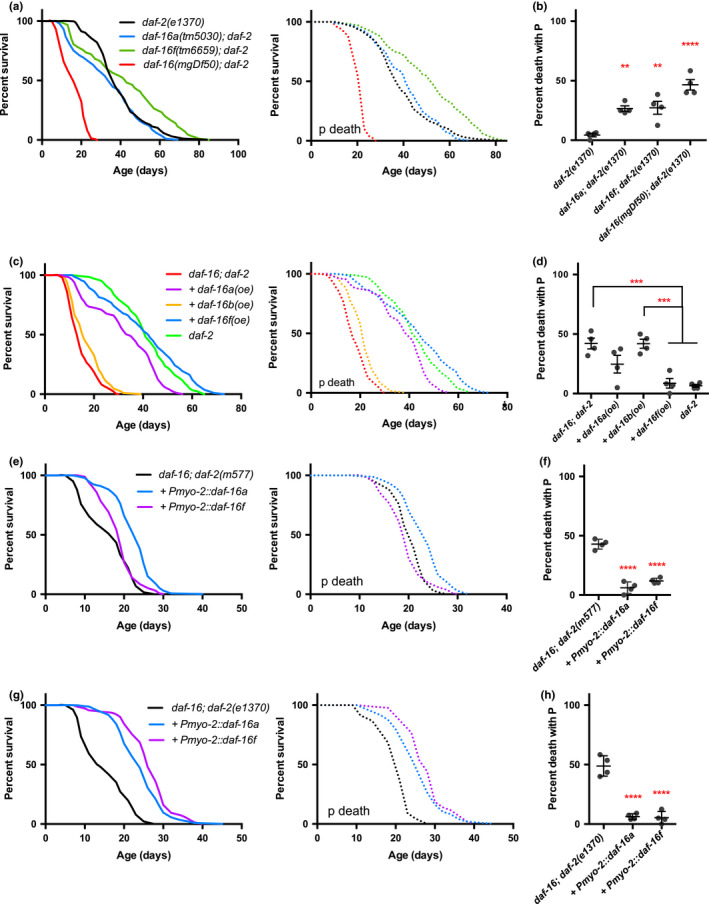
DAF‐16 expression in pharynx suppress P. (a) Lifespan and (b) P frequency of *daf*‐*16* null or isoform‐specific mutant in *daf*‐*2(e1370)* mutant background. *N* = 2 trials. (c) Lifespan and (d) P frequency of isoform‐specific overexpression in *daf*‐*16*;*daf*‐*2(e1370)* double mutant background. *N* = 2 trials. (e) Lifespan and (f) P frequency of *daf*‐*16*;*daf*‐*2(m577)* double mutant overexpressing *daf*‐*16* isoforms in the pharynx. *N* = 2 trials. (g) Lifespan and (H) P frequency of *daf*‐*16*;*daf*‐*2(e1370)* double mutant overexpressing *daf*‐*16* isoforms in the pharynx. *N* = 2 trials

We have described how *daf*‐*2(rf)* increases lifespan by causing p Age and, in some contexts, by reducing P frequency. Both Daf‐c and Age phenotypes of *daf*‐*2* require the DAF‐16 FOXO transcription factor. This raises the possibility that effects of IIS on P and p involve different functions of DAF‐16. We explored this first by testing effects of the null mutation *daf*‐*16(mgDf50)*. In a *daf*‐*2(*+*)* background, *daf*‐*16(0)* reduced mean p lifespan (−16.8%, *p* < 0.0001) and modestly increased P frequency (Figure 4g,h; Appendix S1: Table S8). *daf*‐*2* mutant longevity is to some extent attributable to resistance to *E. coli* infection (Garsin et al., 2003; Podshivalova et al., 2017), suggesting that the p life‐shortening effect of *daf*‐*16(0)* could reflect increased susceptibility to infection. But this is not the case, since *daf*‐*16(0)* shortened mean lifespan more when bacterial proliferation was blocked using antibiotics (−29.9%, *p* < 0.0001) (Appendix S1: Figure S5). We then tested whether the effects of *daf*‐*2(rf)* on both P and p deaths are *daf*‐*16* dependent and found that they were, in both classes of *daf*‐*2* mutant (Figure 5a,b; Appendix S1: Figure S6B, C; Table S9).

*daf*‐*16* isoforms differ in their expression patterns, as do mammalian FOXOs, and also in their effects on *C. elegans* lifespan (Bansal et al., 2014; Chen et al., 2015; Kwon et al., 2010; Lin et al., 2001) (Appendix S1: Figure S6A). P*daf*‐*16a* drives expression in most tissues but not in the pharynx, P*daf*‐*16b* in the pharynx, spermathecae, and neurons, while P*daf*‐*16f* drives expression in almost all tissues including the pharynx (Kwon et al., 2010). At 20˚C in a *daf*‐*16(0)*;*daf*‐*2(e1370)* background, the *Pdaf*‐*16a*::*daf*‐*16a* transgene array *lpIs12* (Kwon et al., 2010) had little effect on P frequency but largely restored p Age, while the *Pdaf*‐*16b*::*daf*‐*16b* transgene array *lpIs13* did not rescue either reduced P or p Age. By contrast, the *Pdaf*‐*16f*::*daf*‐*16f* transgene array *lpIs14* both reduced P frequency and restored p Age (Figure 5c,d; Appendix S1: Table S10).

One possibility is that *daf*‐*16f* but not *daf*‐*16a* expression suppresses P due to the former's pharyngeal expression, rather than to differences in DAF‐16 isoform protein sequence. To test this, we constructed transgenic lines with expression of *daf*‐*16a* or *daf*‐*16f* limited to the pharynx using a *myo*‐*2* promoter (Okkema & Fire, 1994). Pharyngeal overexpression of either DAF‐16A or DAF‐16F was found to reduce P frequency in wild type (Appendix S1: Figure S6E) and in *daf*‐*16*;*daf*‐*2* mutants with both classes of *daf*‐*2* mutation (Figure 5f,h), without reducing pharyngeal pumping rate in early adulthood (Appendix S1: Figure S7). Thus, *daf*‐*16* isoform‐specific effects here are more attributable to spatial differences in expression than in gene‐regulatory specificity.

Restoration of *daf*‐*2* p Age by *Pmyo*‐*2*::*daf*‐*16* proved to be *daf*‐*2* allele specific. *Pmyo*‐*2*::*daf*‐*16a* resulted in p Age in a wild‐type background, and both *daf*‐*16*;*daf*‐*2* backgrounds. By contrast, *Pmyo*‐*2*::*daf*‐*16f* only restored p Age in the *daf*‐*16*;*daf*‐*2(e1370)* background (Figure 5e,g; Appendix S1: Figure S6D; Table S11; Table S12). This could reflect the fact that class 2 but not class 1 *daf*‐*2* mutations cause DAF‐16F nuclear localization, whereas both classes cause DAF‐16A nuclear localization (Bansal et al., 2014).

Taken together, these results imply that *daf*‐*16f* expression in the pharynx protects against P in a tissue‐autonomous fashion. They also suggest that pharyngeal *daf*‐*16* expression promotes p Age, since pharyngeal overexpression of *daf*‐*16* extends lifespan to an extent comparable to that resulting from intestinal overexpression, as previously reported (Alic et al., 2014; Libina et al., 2003). However, there are two caveats with using multicopy transgene arrays to investigate gene function. First, their overexpression can lead to effects that do not reflect wild‐type gene action; this was suggested to be the case for the *Pdaf*‐*16f* transgene array *lpIs14*, which leads to a fourfold increase in mRNA compared with wild type (Bansal et al., 2014; Chen et al., 2015). Second, transgenes expressed from the cloned upstream promoter may not capture the full function of the isoform; this was shown for *daf*‐*16a*, which can be expressed from the *daf*‐*16f* promoter via alternative splicing (Chen et al., 2015).

To further investigate the role of *daf*‐*16a* and *daf*‐*16f* in IIS effects on P and p death while excluding these possible sources of artifact, we used the *daf*‐*16a(tm5030)* and *daf*‐*16f(tm6659)* isoform‐specific mutations which selectively abrogate expression of the isoform affected without changing mRNA levels of the other isoforms (Chen et al., 2015). While neither mutant increased P frequency in an otherwise wild‐type background (Appendix S1: Figure S6G), abrogation of either isoform restored wild‐type P frequency in *daf*‐*2(1370)* (Figure 5b; Appendix S1: Table S9). These results are consistent with pharyngeal expression of *daf*‐*16* from the *daf*‐*16f* promoter protecting against P. Given that *daf*‐*16a* is expressed from the *daf*‐*16f* promoter, we postulate that at endogenous levels, *Pdaf*‐*16f*‐mediated expression in the pharynx of both *daf*‐*16f* and *daf*‐*16a* is required to reduce P. Here, the restoration of P by *daf*‐*16f* is particularly notable, since this mutation was previously found not to suppress *daf*‐*2* Daf‐c, Age, or stress resistance (Chen et al., 2015). Thus, *daf*‐*16f* plays a more important role in preventing P than in other *daf*‐*2* traits, including p Age.

*daf*‐*16a(tm5030)* but not *daf*‐*16f(tm6659)* partially suppressed class 1 *daf*‐*2(m577)* Age, consistent with previous observations (Chen et al., 2015), and the effect of *daf*‐*16a(tm5030)* was due to reduced p Age (Appendix S1: Figure S6B, C). Unexpectedly, *daf*‐*16f(tm6659)* modestly enhanced *daf*‐*2(e1370)* Age and markedly increased p Age (mean lifespan +28.7; *p* < 0.0001) (Figure 5a; Appendix S1: Table S9). A previous study using *e1370* and the class 1 allele *daf*‐*2(e1368)* (which is similar to *m577*) saw no effect of *daf*‐*16f(tm6659)* on lifespan (Chen et al., 2015); the difference between our results could reflect the use in the prior study of the drug 5‐fluoro‐2′‐deoxyuridine (FUDR) to block progeny production. FUDR significantly extends *daf*‐*2(e1370)* lifespan at 20˚C (+19%, *p* < 0.0001) (Appendix S1: Figure S8) (Anderson et al., 2016). This could imply the presence of mildly life‐limiting pathologies caused by *daf*‐*2(e1370)* that are suppressed by both *daf*‐*16f(tm6659)* and FUDR.

### 2.6 Opposing effects on *daf*‐*2* Age by *daf*‐*12* are due to increased P and suppression of bagging

**Figure 6:**
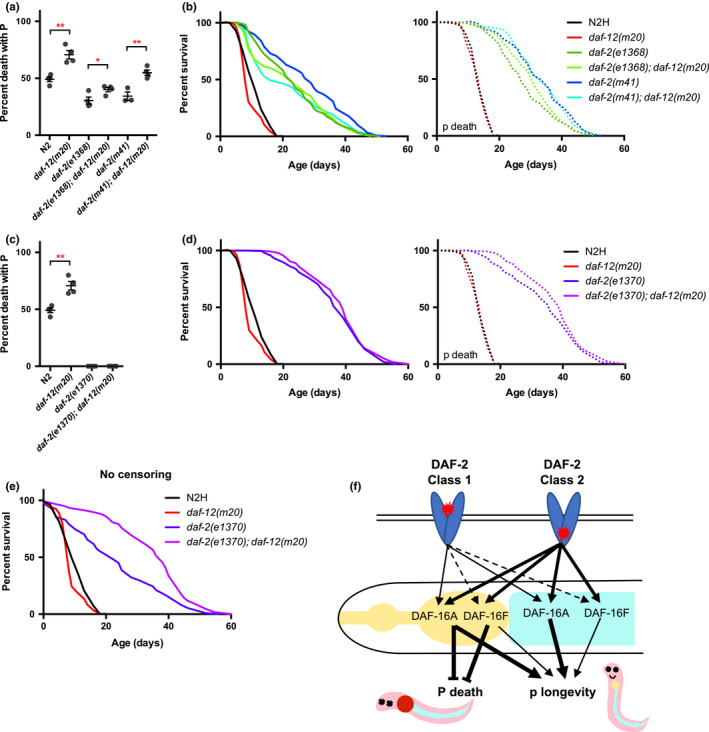
Effect of *daf*‐*12(m20)* mutation on *daf*‐*2* Age at 25˚C, and model of the effects of *daf*‐*2* mutations on *C.elegans* lifespan. (a) P frequency and (b) lifespan of wild type and two class 1 *daf*‐*2* mutants without or with *daf*‐*12(m20)* at 25˚C, with internal hatching censored. *daf*‐*12(m20)* did not significantly affect p Age in class 1 *daf*‐*2* mutant. *N* = 2 trials. (c) P frequency and (d, e) lifespan of wild type and class 2 *daf*‐*2(e1370)* mutant without or with *daf*‐*12(m20)* at 25˚C, with internal hatching censored (d) or not censored (e) after necropsy. *daf*‐*12(m20)* did not significantly affect lifespan in class 2 *daf*‐*2* mutant. *N* = 2 trials. (f) Model for how class 1 and class 2 *daf*‐*2* mutations affect *C. elegans* lifespan. Thickness of lines from DAF‐2 receptors represent severity of mutant effect. Red stars represent the lesion; in most cases, class 1 and class 2 mutations affect the extracellular and intracellular regions of the DAF‐2 receptor, respectively (Kimura et al., 1997; Patel et al., 2008). Class 1 and class 2 mutations exert differential effects on the two major isoforms of DAF‐16 in the pharynx and the intestine. Dashed lines indicate potential effect

Combining two mutations affecting lifespan can produce complex effects that are difficult to interpret, as in the case of *daf*‐*2* and the *daf*‐*12* steroid hormone receptor (Larsen et al., 1995). One difference between the *daf*‐*2* mutant classes is that *daf*‐*12* partially suppresses Age in class 1 mutants, but can enhance it in class 2 mutants (Gems et al., 1998; Patel et al., 2008). How DAF‐12 signaling can have such opposite effects is a long‐standing puzzle. To understand the effects of *daf*‐*12* on the two classes of *daf*‐*2* mutants in terms of effects on P and p deaths, we performed mortality deconvolution on *daf*‐*12(m20)* in wild‐type and *daf*‐*2* mutant backgrounds. Trials were performed at 25˚C and the class 1 allele *daf*‐*2(m41)* and class 2 allele *daf*‐*2(e1370)* were included, to be comparable to the original study in which effects on lifespan of allele class‐specific interactions between *daf*‐*2* and *daf*‐*12* were described (Larsen et al., 1995); we also included an additional class 1 allele, *daf*‐*2(e1368)*. We recently reported that, when confounding effects of *fln*‐*2* are excluded, *daf*‐*12(m20)* modestly reduces *daf*‐*2(m41)* lifespan by increasing P death frequency (Zhao et al., 2019). In the present study, necropsy analysis revealed that *daf*‐*12(m20)* increases P frequency in both wild type and class 1 but not class 2 *daf*‐*2* mutant backgrounds (Figure 6a–c).

An unexpected bonus of necropsy analysis using high magnification microscopy was better detection of internal hatching, specifically of dauer larvae which are difficult to observe within corpses since they are relatively immobile. Consistent with the previous observation of late progeny production by class 2 but not class 1 *daf*‐*2* mutants (Gems et al., 1998), 18% of the corpses of *daf*‐*2(e1370)* mutants dying after day 10 were found to contain live dauer larvae (Appendix S1: Figure S9), but none of the *daf*‐*2(e1368)* or *daf*‐*2(m41)* mutants. By contrast, internally hatched dauer larvae were not seen in *daf*‐*2(e1370)*;*daf*‐*12* mutants, nor do they produce late progeny (Gems et al., 1998; Larsen et al., 1995; Vowels & Thomas, 1992).

Strikingly, when worms with internally hatched dauer larvae were censored, *daf*‐*12(m20)* no longer enhanced *daf*‐*2(e1370)* Age (Figure 6d,e; Appendix S1: Table S13). We conclude that *daf*‐*12(m20)* weakly suppresses *daf*‐*2* Age by increasing P frequency, and that this effect is limited to class 1 *daf*‐*2* mutants since P death is fully suppressed in class 2 mutants; and that *daf*‐*12(m20)* has little effect on *daf*‐*2* p Age in either mutant class if corpses with internally hatched larvae are censored. This resolves the old puzzle of how *daf*‐*12* can produce opposite effects on *daf*‐*2* Age.

## 3 DISCUSSION

In this study, we have applied the new technique of mortality deconvolution to discover how effects of IIS on survival result from alteration of P and p death. The results provide new insights into earlier findings relating to the genetics of lifespan (Chen et al., 2015; D. Gems et al., 1998; Larsen et al., 1995; Nanji et al., 2005; Patel et al., 2008; Tawo et al., 2017). IIS pathway mutations strongly increase p lifespan, but in some cases also reduce P frequency in a *daf*‐*2* allele‐ and temperature‐dependent manner. We present evidence that the reduction in P frequency results from expression from *Pdaf*‐*16f* in the pharynx of *daf*‐*16f* and probably also *daf*‐*16a*. Our findings identify the pharynx as a major tissue where DAF‐16 functions to regulate nematode lifespan. We also explain away a quarter‐century‐old enigma involving the effects on aging of allele‐specific interactions between *daf*‐*2* and *daf*‐*12*. These new insights demonstrate the utility of mortality deconvolution as a technique for understanding the genetics of aging in *C. elegans*.

### 3.1 Distinct effects of IIS mutants on P and p

The large effects on lifespan of mutations in genes such as *daf*‐*2* and *age*‐*1* could imply action on core mechanisms of aging that affect all life‐limiting senescent pathology. Alternatively, IIS could act in different ways on different causes of death. Our findings presented here are consistent with the latter scenario, since IIS exerts differential effects on P and p death in a way that involves differential expression of *daf*‐*16*, both spatially and in terms of the *daf*‐*16* isoforms involved (summarized in Figure 6f).

All *daf*‐*2* and *age*‐*1* mutants examined exhibited p Age, but marked reduction in P frequency was largely restricted to class 2 *daf*‐*2* mutants. Like Eat and Unc (Gems et al., 1998), abrogation of P death is a temperature‐sensitive pleiotropic trait of class 2 *daf*‐*2* mutants, such that P death is reduced at 15˚C and 20˚C, but absent at 25˚C (Figure 2). Given that mutation of *eat*‐*2* reduces both pharyngeal pumping rate and P death (Zhao et al., 2017), it seems likely that cessation of pharyngeal pumping at 25˚C contributes to the temperature‐sensitive abrogation of P death in class 2 mutants. The fact that P frequency is also reduced at lower temperatures where pumping rate is normal (Figure 3a) implies the action of other protective mechanisms. Possibilities here are a more robust pharyngeal cuticle, consistent with elevated expression of extracellular matrix genes in *daf*‐*2* mutants (Ewald et al., 2015), and enhancement of innate immunity, which is well documented in *daf*‐*2* mutants (Evans et al., 2008; Garsin et al., 2003).

### 3.2 Distinct action of *daf*‐*16* isoforms on P and p

Aging is a phenomenon of many diseases. It is therefore surprising that mutation of single genes such as *daf*‐*2* is able so potently to delay the whole aging process. Our findings present an emerging picture in which *daf*‐*2* acts via different *daf*‐*16* isoforms in different tissues to suppress distinct pathologies, here the two forms of death, P and p. Overexpression of *daf*‐*16f* but not *daf*‐*16a* under their endogenous promoters reduced P frequency in *daf*‐*16(0)*;*daf*‐*2(e1370)* worms (Figure 5d). Moreover, pharynx‐limited expression of either *daf*‐*16a* or *daf*‐*16f* reduced P frequency (Figure 5f,h). This implies that expression of DAF‐16 in the pharynx from *Pdaf*‐*16f* prevents P death. However, while DAF‐16F driven by its endogenous promoter is implicated in prevention of P death, it is likely that DAF‐16A also plays a role, since genetic abrogation of either *daf*‐*16a* or *daf*‐*16f* restored high P frequency in *daf*‐*2(1370)* mutants (Figure 5b). Consistent with this, *daf*‐*16a* has been shown to be also expressed from the *daf*‐*2f* promoter (Chen et al., 2015). Importantly, restoration of P in *daf*‐*2(e1370)* mutants by mutation of *daf*‐*16f* shows that the relative importance of *daf*‐*16f* compared with *daf*‐*16A* is greater for reducing P than for p Age, Daf‐c, and stress resistance (Chen et al., 2015) (Figure 5b, Figure 6f). This suggests that a major role of *daf*‐*16f* is protection against pharyngeal senescence.

The unexpected finding that pharynx‐limited *daf*‐*16* rescue promotes *daf*‐*2(e1370)* p Age (Figure 5g) suggests that the worm pharynx plays a greater role in the regulation of senescence than previously realized. Previous studies established the importance for *daf*‐*2* Age of DAF‐16 in the intestine, and to a smaller extent the neurons, though *daf*‐*16* rescue at each site only partially restored Age in *daf*‐*16*;*daf*‐*2* mutants (Libina et al., 2003). The role in aging of *daf*‐*16* action in the pharynx, however, has not been investigated until now. Effects of pharyngeal DAF‐16 action on lifespan could operate in a tissue‐autonomous or nonautonomous fashion. The former would imply that pharyngeal pathology is life limiting. The latter scenario (which seems more plausible) would be consistent with established tissue‐nonautonomous effects of DAF‐16 on longevity and tumorigenesis, from the intestine and the hypodermis, respectively (Alic et al., 2014; Qi et al., 2012) (Figure 6f).

Phenotypic differences between *daf*‐*2* alleles to some extent reflect the overall level of receptor impairment. However, *daf*‐*2* mutants do not fall into a simple allelic series in terms of severity, and the existence of two classes of mutant suggests differential impairment of distinct elements of DAF‐2 receptor function (Gems et al., 1998). These elements appear to correspond to signaling through the PI3 kinase and Ras pathways (Nanji et al., 2005). Analysis of human insulin receptor mutants with lesions equivalent to those in *daf*‐*2* alleles suggests that class 1 mutations may lead to reduced receptor protein levels, while class 2 alleles may cause a relatively greater decrease in Ras pathway signaling (Patel et al., 2008). Taken together with new findings presented here, this suggests that greater reduction in Ras signaling enhances pharyngeal resistance to infection in a manner dependent on pharyngeal action of DAF‐16. One possibility is that the increased DAF‐2 protein levels resulting from *daf*‐*2(gk390525)* (Tawo et al., 2017) cause a relatively greater increase in Ras signaling, thus accounting for the resulting increase in P death frequency.

### 3.3 *daf*‐*2 daf*‐*12* interactions: explaining away an old conundrum

The *daf*‐*16* dependence of *daf*‐*2* Age (Kenyon et al., 1993) was an early illustration of the power of epistasis analysis applied to lifespan genetics. However, in many subsequent studies, combined effects of interventions affecting lifespan yielded results which, though intriguing, were hard to interpret (Gems et al., 2002). For example, *daf*‐*12(m20)* partially suppressed *daf*‐*2(m41)* Age, but enhanced *daf*‐*2(e1370)* Age, a somewhat paradoxical finding hinting at interesting and complex underlying molecular interactions between IIS and steroid signaling affecting aging (Gems et al., 1998; Larsen et al., 1995). However, application of mortality deconvolution analysis has yielded an explanation for *daf*‐*2 daf*‐*12* interactions that is more mundane, if somewhat complex.

First, as described previously, mortality deconvolution analysis led to the discovery that the widely used N2 male stock (N2M) distributed by the *Caenorhabditis* Genetics Center, carries an X‐linked *fln*‐*2(ot611)* mutation that increases lifespan by reducing P frequency (Zhao et al., 2019). This mutation confounded previous comparisons of *daf*‐*2*;*fln*‐*2*+ vs *daf*‐*2*; +*daf*‐*12* leading to a false impression that *daf*‐*12(m20)* can substantially suppress *daf*‐*2* class 1 Age. However, in the absence of *fln*‐*2*, *daf*‐*12* does still increase P frequency to a smaller extent, and by doing this weakly suppresses class 1 *daf*‐*2* Age (Figure 6a,b) (Zhao et al., 2019). Suppression of class 1 *daf*‐*2* Age by *daf*‐*12* due solely to increased P frequency is consistent with neck‐and‐shoulder type survival curves seen previously (Larsen, 1993) (Figure 3b in that study).

Second, in the case of class 2 *daf*‐*2* mutants, necropsy analysis revealed the presence of a proportion of adults with internally hatched larvae which had formed dauers. When these were censored (removed from survival curves), *daf*‐*12(m20)* did not enhance *daf*‐*2* Age (Figure 6d,e). This suggests that the previously observed enhancement by *daf*‐*12* of class 2 *daf*‐*2* Age (Gems et al., 1998; Larsen et al., 1995; McCulloch & Gems, 2007) was due to prevention of death due to internal hatching in *daf*‐*2(e1370)* mutants. Death from internal hatching is caused by larvae devouring their mother from within; it is unclear how the presence of dauers, which are non‐feeding, increases maternal mortality. Possibilities are tissue damage from matriphagic activity by pre‐dauer stages, and secretions by mature dauers. It was previously reported that *daf*‐*12(m20)* strongly suppresses late progeny production and death by internal hatching in *daf*‐*2(e1370)* (Gems et al., 1998; Larsen et al., 1995). The mechanism by which *daf*‐*12* prevents egg retention in *daf*‐*2* mutants remains unknown.

Results presented here show that in the absence of internal hatching *daf*‐*12(m20)* has no impact on p Age in either class of *daf*‐*2* mutant. For class 2 mutants, this is consistent with several previous reports in which *daf*‐*12* did not enhance *daf*‐*2(e1370)* Age in contexts where internal hatching does not occur: in hermaphrodites treated with FUDR (Dumas et al., 2013), in axenic culture (Vanfleteren & Braeckman, 1999), and in males (McCulloch & Gems, 2007).

For class 1 mutants, published findings imply a more complex story. A lack of effect on p Age predicts that *daf*‐*12* will not decrease maximum lifespan in class 1 *daf*‐*2* mutants. That was observed for *daf*‐*2(m41)* in one study using conditions similar to those used here (25˚C, NGM plates) (Larsen, 1993). However, in another study using monoxenic liquid culture at 22.5˚C, *daf*‐*2(m41)* Age was fully suppressed by *daf*‐*12(m20)* (McCulloch & Gems, 2007), and in a third which used culture on NGM plates at 15˚C or 22.5˚C, maximum lifespan was reduced in most cases, though not that of *daf*‐*2(m41)* (Gems et al., 1998). This suggests that under different conditions to those used here, and in different class 1 alleles to those tested here, class 1 *daf*‐*2* p Age can become partially or even fully *daf*‐*12* dependent. Thus, some unresolved complexities remain.

A further consideration is that the canonical *daf*‐*12(m20)* allele used here is not nullimorphic. The *daf*‐*12(rh61rh411)* null allele shortens mean lifespan considerably more than *m20*, though it did not reduce maximum lifespan in several previous reports (Fisher & Lithgow, 2006; Gerisch et al., 2001). This lifespan shortening is partly due to a large increase in the frequency of P death (+59%, *p* = 0.0001), but also a significant reduction in p lifespan (−23%, *p* < 0.0001) (Appendix S1: Figure S10; Table S14).

These findings illustrate how the meaning of complex genetic phenomena, here, the opposite effects of *daf*‐*12* on Age in the two classes of *daf*‐*2* mutant, can sometimes be revealed by means of careful phenotypic analysis rather than molecular genetic investigation. More broadly, they illustrate the importance in understanding the genetic control of aging of discovering how the multiple pathologies that senescence is comprised of are determined.

## 4 EXPERIMENTAL PROCEDURES

### 4.1 Culture conditions and strains

*Caenorhabditis elegans* were maintained using standard protocols (Brenner, 1974). Unless otherwise stated, all strains were grown at 20˚C on nematode growth media (NGM) with plates seeded with *E. coli* OP50 to provide a food source. An N2 hermaphrodite stock recently obtained from the Caenorhabditis Genetics Center (CGC) was used as wild type (N2H). All strains were checked for the *fln*‐*2* genotype. For those found to contain the *fln*‐*2(ot611)* background mutation derived from the CGC N2 male stock (Zhao et al., 2019), strains were outcrossed with N2H.

*Caenorhabditis elegans* strains used included the following. CB1375 *daf*‐*18(e1375)*, DR20 *daf*‐*12(m20)*, DR1296 *daf*‐*2(e1370)*;*daf*‐*12(m20)*, DR1547 *daf*‐*2(m41)*;*daf*‐*12(m20)*, FX5030 *daf*‐*16(tm5030)*, FX6659 *daf*‐*16(tm6659)*, GA91 *daf*‐*16(mgDf50)*;*daf*‐*2(m577)*, GA312 *daf*‐*2(e1368)*;*daf*‐*12(m20)*, GA1062 *muEx211 [pNL213(ges*‐*1p*::*GFP*::*daf*‐*16)*+*rol*‐*6]*, GA1063 *daf*‐*16(mu86) muEx211*, GA1928 *daf*‐*2(e1370)*, GA1945 *daf*‐*2(m41)*, GA1946 *wuEx304 [Pmyo*‐*2*::*daf*‐*16a*+*rol*‐*6(su1006)]*, GA1948 *wuEx305 [Pmyo*‐*2*::*daf*‐*16f*+*rol*‐*6(su1006)]*, GA1952 *daf*‐*16(mgDf50)*, GA1957 *daf*‐*2(m120)*, GA1958 *daf*‐*2(m596)*, GA1959 *daf*‐*2(m577)*, GA1960 *daf*‐*2(e1368)*, GA1990 *daf*‐*2(gk390525)*, GR1168 *age*‐*1(mg44)*/*mnC1 [dpy*‐*10(e128) unc*‐*52(e444)]*, HT1881 *daf*‐*16(mgDf50)*;*daf*‐*2(e1370) unc*‐*119(ed3)*;*lpIs12 [daf*‐*16a*::*RFP*+*unc*‐*119(*+*)]*, HT1882 *daf*‐*16(mgDf50)*;*daf*‐*2(e1370) unc*‐*119(ed3)*;*lpIs13 [daf*‐*16b*::*CFP*+*unc*‐*119(*+*)]*, HT1883 *daf*‐*16(mgDf50)*;*daf*‐*2(e1370) unc*‐*119(ed3)*;*lpIs14 [daf*‐*16f*::*GFP*+*unc*‐*119(*+*)]*, HT1890 *daf*‐*16(mgDf50)*;*daf*‐*2(e1370)*, TJ1052 *age*‐*1(hx546)*.

### 4.2 Construction of transgenic *C. elegans* strains

cDNA containing the coding regions of *daf*‐*16a* and *daf*‐*16f* were cloned into the L3790 vector (Addgene plasmid # 1596) containing the pharyngeal muscle‐expressed promoter *Pmyo*‐*2*. The resulting plasmids were injected using standard means at a concentration of 10 ng/μl into the gonad syncytium of wild‐type adult hermaphrodites, using a *rol*‐*6(su1006)* plasmid as a co‐injection marker to obtain roller lines with heritable extrachromosomal transgene arrays.

### 4.3 Lifespan and necropsy analysis (mortality deconvolution)

Unless otherwise specified, lifespan assays were carried out at 20˚C and without the use of 5‐fluoro‐2′‐deoxyuridine (FUDR). At the start of the assay, L4 animals were transferred to NGM plates seeded two days before with *E. coli* OP50, and transferred daily during the egg‐laying period or every 3–7 days thereafter. Worms were scored as dead when they fail to respond to gentle touch with a worm pick. The pharyngeal status of corpses (P or p) was scored using the highest magnification of a dissecting microscope or using Nomarski (differential interference contrast [DIC]) microscopy, as described (Zhao et al., 2017). Animals lost due to desiccation, matricidal hatching, or rupture were censored. All lifespan assays were conducted at least twice. Combined data from all trials were used to generate survival plots using the application GraphPad Prism (GraphPad Software, Inc.) unless otherwise specified, and statistical tests for significant difference in survival between populations were performed using the log‐rank test.

In order to estimate the proportion of total Age in *daf*‐*2* mutants attributable to the three deconvolved determinants of overall lifespan (reduced P frequency, P Age, and p Age), we calculated the changes to wild‐type lifespan when each of the determinants changes to the level of the *daf*‐*2* mutants.

For all lifespan experiments involving *daf*‐*2* mutants, animals were raised at 15˚C and shifted to the test temperature at the L4 stage. For carbenicillin treatment, carbenicillin solution was added topically to plates with two‐day‐old *E. coli* lawns to a final concentration of 4 mM, and left to dry overnight.

Maternally rescued *age*‐*1(mg44)* homozygous segregants from GR1168 *age*‐*1(mg44)*/+ mothers were identified by singling L4 s and after 5 days testing for the presence of all dauer progeny (due to absence of maternal rescue) (Gottlieb & Ruvkun, 1994). *mg44* homozygotes were picked onto the lifespan assay plate.

### 4.4 Microscopy

For measuring bacterial invasion of pharyngeal tissue, *E. coli* OP50‐RFP (Zhao et al., 2017) was grown overnight in LB broth supplemented with 25 μg/ml tetracycline, then pelleted and resuspended in OP50 media without tetracycline before seeding. Animals were transferred to NGM plates seeded with OP50‐RFP from L4 and transferred daily until imaging. Worms were then allowed to crawl on an unseeded plate for 5 minutes to remove surface fluorescent bacteria, before being mounted onto 2% agar pads with 0.1% levamisole. They were then viewed using a Zeiss Axioskop 2 plus microscope with a rhodamine filter to determine presence of RFP foci in pharyngeal tissue.

### 4.5 Pharyngeal pumping assays

Animals were examined on NGM plates without disturbance for 30 sec under a dissecting microscope, and the number of pharyngeal pumps scored using a manual click counter and a stopwatch.

### 4.6 Statistical analysis

Survival data were analyzed using GraphPad Prism. For whole populations (including both P and p deaths), animals lost due to causes other than aging were right censored. In the case of P and p subpopulations, deaths due to causes other than aging were excluded from the analysis rather than censored. The non‐parametric log‐rank test was used to detect statistically significant lifespan differences between cohorts. P frequencies between different cohorts were compared by one‐way ANOVA. Sample sizes and statistics for each lifespan are provided in the lifespan tables in the Supplementary Tables. Raw mortality data are provided in Ziehm tables (see Supplementary Data). Pumping rate between different cohorts were compared by two‐way ANOVA with multiple comparisons corrected with the Dunnett's test using GraphPad Prism.

## CONFLICT OF INTEREST

The authors declare no competing interests.

## AUTHOR CONTRIBUTIONS

D. G. and Y. Z. conceived the study. F. A., E. R. G., I. M., H. W., B. Z., and Y. Z. performed the experiments. H. C. provided technical support. D. G. and Y. Z. analyzed and interpreted the data and wrote the manuscript.

## Supporting information

Appendix S1

Data S1

## Data Availability

All statistics relating to survival analysis are provided in the Supplementary tables. Raw mortality data are provided in Ziehm tables in Data S1.
