# Supplementary material for: Mutation of daf‐2 extends lifespan via tissue‐specific effectors that suppress distinct life‐limiting pathologies
Source: Aging Cell. 2021 Feb 20;20(3):e13324. doi: 10.1111/acel.13324 (PMC7963334; doi:10.1111/acel.13324)
Supplement: Supplementary file 1 — Appendix S1 [file ACEL-20-e13324-s001.docx]

Supplementary Material

**Mutation of *daf-2* extends lifespan via tissue-specific effectors that suppress distinct life-limiting pathologies**

Yuan Zhao, Bruce Zhang, Ioan Marcu, Faria Athar, Hongyuan Wang, Evgeniy R. Galimov, Hannah Chapman, David Gems

**Supplementary Figure 1**. Effect of class 1 and class 2 *daf-2* mutations on P and p at 15˚C.

**Supplementary Figure 2**. Comparison between class 1 and class 2 *daf-2* mutants at 20˚C and 25˚C.

**Supplementary Figure 3**. Little effect of *daf-2* RNAi on P frequency.

**Supplementary Figure 4.** Increased P frequency in a *daf-2* gain-of-function mutant.

**Supplementary Figure 5.** *daf-16* null mutations shortens lifespan on non-proliferating bacteria.

**Supplementary Figure 6.** Effect of *daf-16* isoforms on P and p.

**Supplementary Figure 7.** Pharyngeal *daf-16* overexpression does not reduce pharyngeal pumping rate.

**Supplementary Figure 8.** FUDR extends *daf-2(e1370)* lifespan at 20˚C.

**Supplementary Figure 9.** Dauer larvae within *daf-2(e1370)* corpses of adult hermaphrodites that have died of old age.

**Supplementary Figure 10.** *daf-12* null mutation increases P frequency and shortens p lifespan at 20˚C.

**Supplementary Table 1.** Mortality deconvolution of aging *daf-2* mutant populations at 20˚C.

**Supplementary Table 2.** Mortality deconvolution of aging *daf-2* mutant populations at 15˚C.

**Supplementary Table 3.** Mortality deconvolution of aging *daf-2* mutant populations at 25˚C.

**Supplementary Table 4.** Combined mortality deconvolution data for each class of *daf-2* mutant at 20˚C and 25˚C.

**Supplementary Table 5.** Mortality deconvolution of aging *daf-2(gk390525)* gain-of-function mutant populations.

**Supplementary Table 6.** Mortality deconvolution of aging *age-1* mutant populations.

**Supplementary Table 7.** Mortality deconvolution of aging *daf-18* mutant populations.

**Supplementary Table 8.** Mortality deconvolution of aging *daf-16(mgDf50)* null mutant populations in the absence and presence of 4 mM carbenicillin.

**Supplementary Table 9.** Mortality deconvolution of aging *daf-16;* *daf-2* mutant populations.

**Supplementary Table 10.** Mortality deconvolution of aging *daf-16;* *daf-2* mutant populations with overexpression of individual *daf-16* isoforms from their own promoters.

**Supplementary Table 11.** Mortality deconvolution of aging strains with over-expression of individual *daf-16* isoforms in the pharynx.

**Supplementary Table 12.** Mortality deconvolution of aging *daf-16;* *daf-2* mutant populations with overexpression of individual *daf-16* isoforms in the pharynx.

**Supplementary Table 13.** Mortality deconvolution of aging *daf-2; daf-12(m20)* mutant populations at 25˚C.

**Supplementary Table 14.** Mortality deconvolution of aging *daf-12* null mutant populations at 20˚C.

Separate files

**Supplementary Data 1.** Ziehm table with full mortality data (raw data, including censored values, and their causes).

**Supplementary Figure 1**. Effect of class 1 and class 2 *daf-2* mutations on P and p at 15˚C.

(A) Lifespan of the whole population (left) and the p death sub-population (right). (B) Frequency of P deaths.

**Supplementary Figure 2**. Comparison between class 1 and class 2 *daf-2* mutants at 20˚C and 25˚C.

(A) Lifespan and (B) frequency of P deaths of the whole population and the p death sub-population of *daf-2* mutants by class.

**Supplementary Figure 3**. Little effect of *daf-2* RNAi on P frequency.

(A) Lifespan and (B) frequency of P death of *daf-2* RNAi in wild-type from either L4 or the parent generation.

(C) Lifespan and (D) frequency of P death of *daf-2* RNAi in the RNAi-sensitive *rrf-3* mutants. Data from one trial each.

**Supplementary Figure 4**. Increased P frequency in a *daf-2* gain-of-function mutant.

(A) Contribution of increased P frequency and reduced p lifespan to *daf-2(gk390525)* lifespan.

(B) No increase in pumping rate in *daf-2(gk390525)* in early adulthood. A significant decrease in pumping was observed in the mutant on day 6 of adulthood.

(C) Lifespan of *daf-2(gk390525)* on carbenicillin-treated *E. coli*.

**Supplementary Figure 5**. *daf-16* null mutations shortens lifespan on non-proliferating bacteria.

Lifespan of N2 and *daf-16* null mutant on proliferating and carbenicillin-treated non-proliferating *E. coli*.

**Supplementary Figure 6**. Effect of *daf-16* isoforms on P and p.

(A) *daf-16* isoforms and known expression pattern.

(B) Lifespan and (C) P frequency of *daf-16* isoform-specific mutants in a *daf-16(mgDf50); daf-2(m577)* background. N = 2 trials.

(D) Lifespan and (E) P frequency of transgenic lines overexpressing *daf-16a* or *daf-16f* from the exclusively pharynx-expressed promoter *Pmyo-2*. N = 2 trials

(F) Lifespan and (G) P frequency of *daf-16* isoform-specific mutants. N = 2 trials (showing 1 trial).

**Supplementary Figure 7**. Pharyngeal *daf-16* overexpression does not reduce pharyngeal pumping rate.

Pumping rate of *daf-16(mgDf50); daf-2(e1370)* carrying pharyngeal overexpression *daf-16a* or *daf-16f* transgene in early adulthood.

**Supplementary Figure 8**. FUDR extends *daf-2(e1370)* lifespan at 20˚C.

Lifespan of *daf-2(e1370)* on *E. coli* OP50 in the presence or absence of 10μM FUDR. N = 2 trials.

**Supplementary Figure 9**. Dauer larvae within *daf-2(e1370)* corpses of adult hermaphrodites that have died of old age.

Animals were cultured at 25˚C from L4 stage onwards. Nomarski microscopy, 400x magnification.

**Supplementary Figure 10**. *daf-12* null mutation increases P frequency and shortens p lifespan at 20˚C.

(A) Lifespan and (B) P frequency of *daf-12(rh61rh411)* null allele at 20˚C. *daf-12* null shortens lifespan by reducing p lifespan (*p* < 0.0001) and increasing P frequency (*p* = 0.0001). By contrast, *daf-12(m20)* increases P frequency but does not reduce p lifespan.

| **Genotype** | **Number of deaths/ censored** | **%P** | **All deaths** | | | **P deaths** | | | **p deaths** | | | |
| --- | --- | --- | --- | --- | --- | --- | --- | --- | --- | --- | --- | --- |
|  |  |  | **Mean lifespan (days)** | **% change vs. control** | ***p* vs. control (log rank)** | **Mean lifespan (days)** | **% change vs. control** | ***p* vs. control**  **(log rank)** | **Mean lifespan (days)** | **% change vs. control** | ***p* vs. control**  **(log rank)** |  |
| N2 (control) | **[C] 110/14**  [1] 57/6  [2] 53/8 | **46.4**  49.1  43.4 | **17.4**  17.4  17.4 |  |  | **12.6**  13.0  12.2 |  |  | **21.5**  21.6  21.3 |  |  |  |
| *daf-2(e1368)* | **[C] 111/14**  [1] 59/5  [2] 52/9 | **18.9**  22.0  15.4 | **29.2**  26.7  32.1 | **+68.2**  +53.6  +84.8 | **<0.0001**  <0.0001  <0.0001 | **16.8**  17.5  15.8 | **+33.3**  +34.7  +29.4 | **<0.0001**  0.0011  0.0181 | **32.1**  29.3  35.0 | **+49.5**  +35.4  +64.3 | **<0.0001**  <0.0001  <0.0001 |  |
| *daf-2(e1370)* | **[C] 89/39**  [1] 53/15  [2] 36/24 | **3.4**  1.8  5.6 | **41.6**  43.3  39.2 | **+139.6**  +149.1  +125.6 | **<0.0001**  <0.0001  <0.0001 | **29.3**  39.0  24.5 | **+132.7**  +200.8  +101.3 | **0.0011**  0.0296  0.0088 | **42.0**  43.3  40.0 | **+95.7**  +100.5  +87.6 | **<0.0001**  <0.0001  <0.0001 |  |
| *daf-2(m41)* | **[C] 82/36**  [1] 43/15  [2] 39/21 | **22.0**  9.3  35.9 | **27.7**  31.0  24.0 | **+59.4**  +78.5  +38.4 | **<0.0001**  <0.0001  <0.0001 | **15.4**  22.8  13.4 | **+22.5**  +75.5  +9.7 | **0.0183**  0.0007  0.1493 | **31.1**  31.8  30.0 | **+44.9**  +47.3  +40.6 | **<0.0001**  <0.0001  <0.0001 |  |
| *daf-2(m120)* | **[C] 112/10**  [1] 56/6  [2] 56/4 | **4.5**  5.4  3.6 | **48.2**  48.0  48.3 | **+177.4**  +176.4  +178.5 | **<0.0001**  <0.0001  <0.0001 | **17.4**  19.7  14.0 | **+38.0**  +51.7  +15.0 | **0.0065**  0.0114  0.4590 | **49.6**  49.6  49.6 | **+131.0**  +129.4  +132.6 | **<0.0001**  <0.0001  <0.0001 |  |
| *daf-2(m577)* | **[C] 96/21**  [1] 50/7  [2] 46/14 | **29.2**  28.0  30.4 | **22.6**  21.7  23.5 | **+30.1**  +25.2  +35.4 | **<0.0001**  <0.0001  <0.0001 | **13.3**  14.1  12.5 | **+5.7**  +9.1  +2.7 | **0.4108**  0.2943  0.6527 | **26.4**  24.7  28.3 | **+22.9**  +14.2  +32.7 | **<0.0001**  <0.0001  <0.0001 |  |
| *daf-2(m596)* | **[C] 104/10**  [1] 48/6  [2] 56/4 | **11.5**  8.3  14.3 | **35.7**  34.8  36.6 | **+105.7**  +100.1  +110.6 | **<0.0001**  <0.0001  <0.0001 | **21.0**  20.2  21.4 | **+66.6**  +56.2  +75.6 | **<0.0001**  0.0210  0.0014 | **37.6**  36.1  39.1 | **+75.3**  +66.8  +83.2 | **<0.0001**  <0.0001  <0.0001 |  |

Supplementary Table 1. Mortality deconvolution of aging *daf-2* mutant populations at 20˚C.

Worms were maintained at 15˚C and transferred to 20˚C at L4. [C], combined data from all trials, [n], trial number. For P and p subpopulations, animals lost due to internal hatching or vulva rupture were excluded from statistical analysis rather than censored (see Methods). Sample sizes for P and p subpopulations can be estimated from total sample size and %P.

| **Genotype** | **Number of deaths/ censored** | **%P** | **All deaths** | | | **P deaths** | | | **p deaths** | | | |
| --- | --- | --- | --- | --- | --- | --- | --- | --- | --- | --- | --- | --- |
|  |  |  | **Mean lifespan (days)** | **% change vs. control** | ***p* vs. control (log rank)** | **Mean lifespan (days)** | **% change vs. control** | ***p* vs. control**  **(log rank)** | **Mean lifespan (days)** | **% change vs. control** | ***p* vs. control**  **(log rank)** |  |
| N2 (control) | **[C] 101/21**  [1] 55/6  [2] 46/15 | **26.7**  27.3  26.1 | **27.3**  27.4  27.1 |  |  | **22.2**  21.9  22.7 |  |  | **29.1**  29.4  28.7 |  |  |  |
| *daf-2(e1368)* | **[C] 100/20**  [1] 52/7  [2] 48/13 | **12.0**  11.5  12.5 | **36.3**  38.0  34.5 | **+33.2**  +38.9  +27.2 | **<0.0001**  <0.0001  <0.0001 | **29.5**  29.3  30.9 | **+32.8**  +34.1  +29.4 | **0.0115**  0.1676  0.0539 | **37.3**  39.1  35.2 | **+28.0**  +33.0  +22.6 | **<0.0001**  <0.0001  <0.0001 |  |
| *daf-2(e1370)* | **[C] 83/40**  [1] 37/30  [2] 46/10 | **14.5**  13.5  15.2 | **41.3**  39.8  42.4 | **+51.4**  +45.6  +56.4 | **<0.0001**  <0.0001  <0.0001 | **28.8**  27.0  30.1 | **+29.8**  +23.5  +33.0 | **0.0030**  0.0979  0.0206 | **43.4**  41.8  44.6 | **+49.1**  +42.2  +55.5 | **<0.0001**  <0.0001  <0.0001 |  |

Supplementary Table 2. Mortality deconvolution of aging *daf-2* mutant populations at 15˚C.

Worms were maintained and lifespan performed at 15˚C. [C], combined data from all trials, [n], trial number.

| **Genotype** | **Number of deaths/ censored** | **%P** | **All deaths** | | | **P deaths** | | | **p deaths** | | | |
| --- | --- | --- | --- | --- | --- | --- | --- | --- | --- | --- | --- | --- |
|  |  |  | **Mean lifespan (days)** | **% change vs. control** | ***p* vs. control (log rank)** | **Mean lifespan (days)** | **% change vs. control** | ***p* vs. control**  **(log rank)** | **Mean lifespan (days)** | **% change vs. control** | ***p* vs. control**  **(log rank)** |  |
| N2 (control) | **[C] 96/24**  [1] 46/14  [2] 50/10 | **44.8**  50.0  40.0 | **12.8**  11.7  13.7 |  |  | **8.1**  8.6  7.6 |  |  | **16.5**  14.9  17.8 |  |  |  |
| *daf-2(e1368)* | **[C] 97/26**  [1] 47/16  [2] 50/10 | **28.9**  27.7  30.0 | **25.5**  26.4  24.7 | **+99.9**  +124.6  +80.3 | **<0.0001**  <0.0001  <0.0001 | **14.4**  13.1  15.6 | **+77.8**  +52.7  +105.3 | **<0.0001**  0.0001  <0.0001 | **30.0**  31.4  28.7 | **+81.5**  +110.8  +61.0 | **<0.0001**  <0.0001  <0.0001 |  |
| *daf-2(e1370)* | **[C] 53/76**  [1] 26/39  [2] 27/37 | **0**  0  0 | **36.3**  36.2  36.4 | **+184.0**  +208.0  +165.1 | **<0.0001**  <0.0001  <0.0001 |  |  |  | **36.3**  36.2  36.4 | **+119.2**  +142.4  +104.3 | **<0.0001**  <0.0001  <0.0001 |  |
| *daf-2(m41)* | **[C] 88/31**  [1] 47/12  [2] 41/19 | **31.8**  23.4  41.5 | **26.3**  28.9  23.2 | **+105.6**  +146.1  +69.4 | **<0.0001**  <0.0001  <0.0001 | **10.7**  9.8  11.3 | **+32.0**  +14.6  +48.6 | **0.0002**  0.3612  <0.0001 | **33.5**  34.7  31.7 | **+102.6**  +132.8  +78.1 | **<0.0001**  <0.0001  <0.0001 |  |
| *daf-2(m120)* | **[C] 77/50**  [1] 43/24  [2] 34/26 | **0**  0  0 | **35.8**  36.8  34.5 | **+180.1**  +213.4  +151.2 | **<0.0001**  <0.0001  <0.0001 |  |  |  | **35.8**  36.8  34.5 | **+116.1**  +146.7  +93.7 | **<0.0001**  <0.0001  <0.0001 |  |
| *daf-2(m577)* | **[C] 75/54**  [1] 31/38  [2] 44/16 | **33.3**  19.4  43.2 | **25.3**  28.2  23.3 | **+98.5**  +140.4  +70.0 | **<0.0001**  <0.0001  <0.0001 | **12.6**  13.2  12.5 | **+55.7**  +53.7  +64.1 | **<0.0001**  0.0003  <0.0001 | **31.7**  31.8  31.6 | **+91.6**  +113.5  +77.3 | **<0.0001**  <0.0001  <0.0001 |  |
| *daf-2(m596)* | **[C] 100/23**  [1] 54/9  [2] 46/14 | **21.0**  14.8  28.3 | **29.7**  32.5  26.5 | **+132.9**  +176.7  +93.3 | **<0.0001**  <0.0001  <0.0001 | **14.1**  14.9  13.7 | **+74.3**  +73.7  +80.2 | **<0.0001**  0.0004  <0.0001 | **33.9**  35.5  31.6 | **+104.8**  +138.3  +77.4 | **<0.0001**  <0.0001  <0.0001 |  |

Supplementary Table 3. Mortality deconvolution of aging *daf-2* mutant populations at 25˚C.

Worms were maintained at 15˚C and shifted to 25˚C at L4 stage. [C], combined data from all trials, [n], trial number.

| **Strain, condition** | **Number of deaths/ censored** | **%P** | **All deaths** | | | | | **p deaths** | | | | |
| --- | --- | --- | --- | --- | --- | --- | --- | --- | --- | --- | --- | --- |
|  |  |  | **Mean lifespan (days)** | **% change vs. N2** | ***p* vs. N2**  **(log rank)** | **% change vs.**  **class 1** | ***p* vs. class 1 (log rank)** | **Mean lifespan (days)** | **% change vs. N2** | ***p* vs. N2**  **(log rank)** | **% change vs.**  **class 1** | ***p* vs. class 1 (log rank)** |
| N2  20ºC | **[C] 110/14**  [1] 57/6  [2] 53/8 | **46.4**  49.1  43.4 | **17.4**  17.4  17.4 |  |  |  |  | **21.5**  21.6  21.3 |  |  |  |  |
| Class 1 *daf-2* mutants  20ºC | **[C] 393/81**  [1] 200/33  [2] 193/48 | **20.1**  17.5  22.8 | **29.0**  28.3  29.7 | **+67.0**  +63.0  +71.1 | **<0.0001**  <0.0001  <0.0001 |  |  | **32.3**  30.7  34.1 | **+50.4**  +42.0  +59.6 | **<0.0001**  <0.0001  <0.0001 |  |  |
| Class 2 *daf-2* mutants  20ºC | **[C] 201/49**  [1] 109/21  [2] 92/28 | **4.0**  3.7  4.3 | **45.3**  45.7  44.8 | **+160.8**  +163.4  +157.8 | **<0.0001**  <0.0001  <0.0001 | **+56.2**  +61.6  +50.6 |  | **46.3**  46.6  45.9 | **+115.4**  +115.3  +115.2 | **<0.0001**  <0.0001  <0.0001 | **+43.3**  +51.7  +34.8 | **<0.0001**  <0.0001  <0.0001 |
| N2  25ºC | **[C] 96/24**  [1] 46/14  [2] 50/10 | **44.8**  50.0  40.0 | **12.8**  11.7  13.7 |  |  |  |  | **16.5**  14.9  17.8 |  |  |  |  |
| Class 1 *daf-2* mutants  25ºC | **[C] 360/134**  [1] 179/75  [2] 181/59 | **28.3**  21.2  35.4 | **26.8**  29.2  24.5 | **+110.2**  +148.7  +78.6 | **<0.0001**  <0.0001  <0.0001 |  |  | **32.3**  33.7  30.7 | **+95.5**  +125.9  +72.6 | **<0.0001**  <0.0001  <0.0001 |  |  |
| Class 2 *daf-2* mutants  25ºC | **[C] 130/126**  [1] 69/63  [2] 61/63 | **0**  0  0 | **35.9**  36.6  35.3 | **+181.7**  +211.4  +157.4 | **<0.0001**  <0.0001  <0.0001 | **+34.0**  +25.2  +44.1 | **<0.0001**  0.0025  <0.0001 | **35.9**  36.6  31.6 | **+117.4**  +125.9  +98.4 | **<0.0001**  <0.0001  <0.0001 | **+11.2**  +8.5  +14.9 | **0.0043**  0.2769  0.0051 |

**Supplementary Table 4**. Combined mortality deconvolution data for each class of *daf-2* mutant at 20˚C and 25˚C.

Worms were maintained at 15˚C and shifted to lifespan temperature of either 20ºC or 25ºC at L4 stage. [C], combined data from all trials, [n], trial number.

| **Genotype** | **Number of deaths/ censored** | **%P** | **All deaths** | | | **P deaths** | | | **p deaths** | | | |
| --- | --- | --- | --- | --- | --- | --- | --- | --- | --- | --- | --- | --- |
|  |  |  | **Mean lifespan (days)** | **% change vs. control** | ***p* vs. control (log rank)** | **Mean lifespan (days)** | **% change vs. control** | ***p* vs. control**  **(log rank)** | **Mean lifespan (days)** | **% change vs. control** | ***p* vs. control**  **(log rank)** |  |
| N2 (control) | **[C] 104/16**  [1] 53/8  [2] 51/8 | **38.5**  43.4  33.3 | **18.8**  17.5  20.2 |  |  | **13.0**  12.4  13.7 |  |  | **22.4**  21.3  23.4 |  |  |  |
| *daf-2(gk390525)* | **[C] 124/11**  [1] 62/3  [2] 62/8 | **54.0**  56.5  51.6 | **16.5**  15.8  17.2 | **-12.0**  -9.3  -14.6 | **0.0028**  0.0320  0.0141 | **12.4**  12.4  12.4 | **-4.2**  +0.3  -9.7 | **0.2862**  0.9607  0.0968 | **21.4**  20.2  22.4 | **-4.8**  -5.2  -4.3 | **0.1037**  0.1383  0.2073 |  |

**Supplemental Table 5**. Mortality deconvolution of aging *daf-2(gk390525)* gain-of-function mutant populations.

Trials were performed at 20˚C, without FUDR. [C], combined data from all trials, [n], trial number.

| **Genotype** | **Number of deaths/ censored** | **%P** | **All deaths** | | | **P deaths** | | | **p deaths** | | | |
| --- | --- | --- | --- | --- | --- | --- | --- | --- | --- | --- | --- | --- |
|  |  |  | **Mean lifespan (days)** | **% change vs. control** | ***p* vs. control (log rank)** | **Mean lifespan (days)** | **% change vs. control** | ***p* vs. control**  **(log rank)** | **Mean lifespan (days)** | **% change vs. control** | ***p* vs. control**  **(log rank)** |  |
| N2 (control) | **[C] 145/38**  [1] 51/20  [2] 51/8  [3] 43/10 | **37.2**  35.3  33.3  34.8 | **18.3**  17.0  20.2  17.7 |  |  | **13.2**  13.3  13.7  12.8 |  |  | **21.4**  19.1  23.4  21.6 |  |  |  |
| *age-1(hx546)* | **[C] 106/29**  [1] 60/17  [2] 46/12 | **61.3**  53.3  71.7 | **19.1**  19.6  18.3 | **+4.0**  +15.2  -9.2 | **0.1433**  0.0589  0.8795 | **14.0**  15.0  13.0 | **+5.4**  +12.7  -5.4 | **0.5769**  0.1167  0.1934 | **27.1**  25.0  31.8 | **+27.1**  +30.8  +36.2 | **<0.0001**  0.0011  <0.0001 |  |
| *age-1(mg44)* | **[C] 71/124**  [1] 15/48  [2] 20/55  [3] 35/41 | **43.7**  53.3  50.0  37.1 | **29.1**  23.4  29.2  31.5 | **+58.3**  +37.2  +44.6  +78.1 | **<0.0001**  0.0072  0.0144  <0.0001 | **15.1**  14.5  16.5  14.5 | **+14.3**  +9.2  +20.4  +13.1 | **0.0146**  0.2553  0.0422  0.6638 | **39.8**  32.3  41.8  41.6 | **+86.3**  +68.9  +78.8  +92.7 | **<0.0001**  0.0002  0.0003  <0.0001 |  |

Supplementary Table 6. Mortality deconvolution of aging *age-1* mutant populations.

Trials were performed at 20˚C, no FUDR. [C], combined data from all trials, [n], trial number. As *age-1(mg44)* mutant suffers high proportion of rupture, the proportion of worms which died with swollen pharynx is difficult to measure precisely.

| **Genotype** | **Number of deaths/ censored** | **%P** | **All deaths** | | | **P deaths** | | | **p deaths** | | | |
| --- | --- | --- | --- | --- | --- | --- | --- | --- | --- | --- | --- | --- |
|  |  |  | **Mean lifespan (days)** | **% change vs. control** | ***p* vs. control (log rank)** | **Mean lifespan (days)** | **% change vs. control** | ***p* vs. control**  **(log rank)** | **Mean lifespan (days)** | **% change vs. control** | ***p* vs. control**  **(log rank)** |  |
| N2 (control) | **[C] 102/28**  [1] 51/20  [2] 51/8 | **34.3**  35.3  33.3 | **18.6**  17.0  20.2 |  |  | **13.5**  13.3  13.7 |  |  | **21.3**  19.1  23.4 |  |  |  |
| *daf-18(e1375)* | **[C] 93/42**  [1] 46/29  [2] 47/13 | **20.4**  29.2  10.6 | **12.9**  12.1  13.6 | **-30.9**  -29.1  -32.4 | **<0.0001**  <0.0001  <0.0001 | **11.6**  12.2  10.0 | **-13.7**  -8.0  -27.0 | **0.0600**  0.4926  0.0006 | **13.2**  12.0  14.0 | **-38.1**  -37.0  -39.9 | **<0.0001**  <0.0001  <0.0001 |  |

Supplementary Table 7. Mortality deconvolution of aging *daf-18* mutant populations.

Trials were performed at 20˚C, without FUDR. [C], combined data from all trials, [n], trial number.

| **Genotype** | **Number of deaths/ censored** | **%P** | **All deaths** | | | **P deaths** | | | **p deaths** | | |
| --- | --- | --- | --- | --- | --- | --- | --- | --- | --- | --- | --- |
|  |  |  | **Mean lifespan (days)** | **% change vs. control** | ***p* vs. control (log rank)** | **Mean lifespan (days)** | **% change vs. control** | ***p* vs. control**  **(log rank)** | **Mean lifespan (days)** | **% change vs. control** | ***p* vs. control**  **(log rank)** |
| N2 (control) | **[C] 96/10**  [1] 54/2  [2] 42/8 | **35.4**  33.3  38.1 | **17.6**  18.4  16.6 |  |  | **11.8**  12.0  11.6 |  |  | **20.8**  21.7  19.7 |  |  |
| *daf-16(mgDf50)* | **[C] 126/10**  [1] 74/6  [2] 52/4 | **46.0**  41.9  51.9 | **13.9**  14.3  13.4 | **-21.0**  -22.6  -19.1 | **<0.0001**  <0.0001  0.0010 | **9.9**  9.9  10.0 | **-15.9**  -17.5  -14.0 | **<0.0001**  0.0002  0.0163 | **17.3**  17.4  17.2 | **-16.8**  -19.5  -12.9 | **<0.0001**  <0.0001  0.0124 |
| N2 + carbenicillin (control) | **[C] 118/2**  [1] 68/1  [2] 50/1 | **0**  0  0 | **30.5**  30.6  30.4 |  |  |  |  |  | **30.5**  30.6  30.4 |  |  |
| *daf-16(mgDf50)* + carbenicillin | **[C] 129/8**  [1] 69/7  [2] 60/1 | **0**  0  0 | **21.4**  19.4  23.7 | **-29.9**  -36.6  -22.0 | **<0.0001**  <0.0001  <0.0001 |  |  | **<0.0001**  <0.0001  <0.0001 | **29.9**  19.4  23.7 | **-29.9**  -36.6  -22.0 | **<0.0001**  <0.0001  <0.0001 |

Supplementary Table 8. Mortality deconvolution of aging *daf-16(mgDf50)* null mutant populations in the absence and presence of 4 mM carbenicillin.

Trials were performed at 20˚C, with no FUDR. [C], combined data from all trials, [n], trial number.

| **Genotype** | **Number of deaths/ censored** | **%P** | **All deaths** | | | **P deaths** | | | **p deaths** | | | |
| --- | --- | --- | --- | --- | --- | --- | --- | --- | --- | --- | --- | --- |
|  |  |  | **Mean lifespan (days)** | **% change vs. control** | ***p* vs. control (log rank)** | **Mean lifespan (days)** | **% change vs. control** | ***p* vs. control**  **(log rank)** | **Mean lifespan (days)** | **% change vs. control** | ***p* vs. control**  **(log rank)** |  |
| *daf-2(m577)*  (control) | **[C] 94/28**  [1] 46/14  [2] 48/14 | **33.0**  30.4  35.4 | **23.4**  23.5  23.3 |  |  | **13.8**  12.5  14.6 |  |  | **28.2**  28.3  28.1 |  |  |  |
| *daf-16a(tm5030); daf-2(m577)* | **[C] 99/25**  [1] 53/7  [2] 46/18 | **43.4**  43.4  43.5 | **18.7**  18.1  19.4 | **-20.1**  -23.1  -16.8 | **<0.0001**  0.0001  0.0005 | **12.1**  11.3  13.0 | **-11.8**  -9.9  -11.2 | **0.0113**  0.1420  0.0415 | **23.8**  23.3  24.3 | **-15.7**  -17.7  -13.3 | **<0.0001**  0.0004  0.0001 |  |
| *daf-16f(tm6659); daf-2(m577)* | **[C] 95/20**  [1] 50/10  [2] 45/10 | **30.5**  28.0  33.3 | **24.2**  23.8  24.6 | **+3.4**  +1.4  +5.5 | **0.9013**  0.6853  0.2225 | **14.6**  13.5  15.5 | **+6.4**  +8.0  +6.1 | **0.3597**  0.1350  0.5253 | **28.5**  27.9  29.2 | **+0.9**  -1.6  +3.8 | **0.8314**  0.4162  0.1695 |  |
| *daf-16(mgDf50); daf-2(m577)* | **[C] 102/18**  [1] 51/9  [2] 51/9 | **43.1**  41.2  47.1 | **16.9**  17.1  16.8 | **-27.6**  -27.3  -28.0 | **<0.0001**  <0.0001  <0.0001 | **11.1**  10.7  11.5 | **-18.9**  -14.7  -21.6 | **0.0004**  0.1572  0.0030 | **21.4**  21.6  21.2 | **-24.2**  -23.8  -24.6 | **<0.0001**  <0.0001  <0.0001 |  |
| *daf-2(e1370)*  (control) | **[C] 70/43**  [1] 36/24  [2] 34/19 | **5.7**  5.6  5.9 | **39.1**  39.2  39.1 |  |  | **25.0**  24.5  25.5 |  |  | **40.0**  40.0  39.9 |  |  |  |
| *daf-16a(tm5030); daf-2(e1370)* | **[C] 89/30**  [1] 48/12  [2] 41/18 | **28.1**  27.1  29.3 | **33.5**  33.4  33.6 | **-14.4**  -14.7  -14.1 | **0.1357**  0.2965  0.3050 | **13.8**  13.6  14.1 | **-44.6**  -44.4  -44.8 | **0.0051**  0.0766  0.0497 | **41.2**  40.7  41.7 | **+2.9**  +1.8  +4.3 | **0.8234**  0.8983  0.8076 |  |
| *daf-16f(tm6659); daf-2(e1370)* | **[C] 89/19**  [1] 40/13  [2] 49/6 | **28.1**  20.0  34.7 | **42.2**  44.8  40.2 | **+7.9**  +14.4  +2.7 | **0.0272**  0.0274  0.2563 | **18.6**  19.8  18.1 | **-25.4**  -19.4  -29.0 | **0.2640**  0.5022  0.3306 | **51.5**  51.1  51.9 | **+28.7**  +27.6  +29.8 | **<0.0001**  0.0042  0.0014 |  |
| *daf-16(mgDf50); daf-2(e1370)* | **[C] 96/26**  [1] 46/14  [2] 50/12 | **47.9**  52.2  45.1 | **16.0**  15.3  16.7 | **-59.0**  -60.8  -57.9 | **<0.0001**  <0.0001  <0.0001 | **11.1**  10.8  11.3 | **-55.7**  -55.8  -55.6 | **0.0011**  0.0324  0.0228 | **20.5**  20.3  20.9 | **-48.5**  -49.4  -48.4 | **<0.0001**  <0.0001  <0.0001 |  |

**Supplementary Table 9.** Mortality deconvolution of aging *daf-16;* *daf-2* mutant populations.

Worms were maintained at 15˚C and transferred to 20˚C at L4, no FUDR. [C], combined data from all trials, [n], trial number.

| **Genotype** | **Number of deaths/ censored** | **%P** | **All death** | | | **P death** | | | **p death** | | |
| --- | --- | --- | --- | --- | --- | --- | --- | --- | --- | --- | --- |
|  |  |  | **Mean lifespan (days)** | **% change vs. control** | ***p* vs. control (log rank)** | **Mean lifespan (days)** | **% change vs. control** | ***p* vs. control**  **(log rank)** | **Mean lifespan (days)** | **% change vs. control** | ***p* vs. control**  **(log rank)** |
| *daf-16(mgDf50); daf-2(e1370)*  (control) | **[C] 159/37**  [1] 59/1  [2] 57/3  [3] 43/33 | **45.3**  45.8  52.6  34.9 | **14.7**  16.5  15.1  13.0 |  |  | **11.2**  11.8  11.2  10.3 |  |  | **17.6**  20.4  19.4  14.4 |  |  |
| *daf-16; daf-2(e1370) unc-119(ed3); lpIs12 [daf-16a::RFP + unc-119(+)]* | **[C] 97/127**  [1] 26/32  [2] 32/63  [3] 39/32 | **26.8**  34.6  37.5  12.8 | **32.2**  30.9  30.9  34.0 | **+114.0**  +87.7  +105.1  +162.0 | **<0.0001**  <0.0001  <0.0001  <0.0001 | **17.8**  17.3  19.5  14.4 | **+58.3**  +47.2  +74.6  +39.4 | **<0.0001**  0.0029  0.0009  0.1173 | **37.4**  38.1  37.8  36.9 | **+105.9**  +86.5  +94.5  +156.3 | **<0.0001**  <0.0001  <0.0001  <0.0001 |
| *daf-16; daf-2(e1370) unc-119(ed3); lpIs13 [daf-16b::CFP + unc-119(+)]* | **[C] 120/59**  [1] 39/13  [2] 36/15  [3] 45/31 | **43.3**  46.2  50.0  35.6 | **17.3**  18.2  17.2  16.6 | **+15.0**  +10.2  +14.3  +27.7 | **0.0029**  0.1513  0.0859  0.0004 | **12.1**  11.4  13.7  11.2 | **+8.1**  -2.8  +22.4  +8.3 | **0.0154**  0.9955  0.0009  0.1010 | **21.2**  23.9  20.8  19.6 | **+16.8**  +17.0  +7.1  +35.8 | **0.0017**  0.0354  0.2955  <0.0001 |
| *daf-16; daf-2(e1370) unc-119(ed3); lpIs14 [daf-16f::GFP + unc-119(+)]* | **[C] 112/84**  [1] 36/13  [2] 49/43  [3] 27/28 | **10.7**  19.4  8.2  3.7 | **41.3**  36.4  46.6  38.4 | **+175.0**  +120.7  +208.9  +196.3 | **<0.0001**  <0.0001  <0.0001  <0.0001 | **24.0**  28.0  18.5  18.0 | **+113.9**  +137.7  +65.7  +74.2 | **<0.0001**  0.0002  0.0019  0.0251 | **43.4**  38.4  49.0  39.2 | **+138.8**  +87.8  +152.7  +172.6 | **<0.0001**  <0.0001  <0.0001  <0.0001 |
| *daf-2(e1370)* | **[C] 160/54**  [1] 68/13  [2] 45/12  [3] 47/29 | **5.6**  5.9  4.4  6.4 | **41.6**  42.4  40.1  42.0 | **+177.2**  +157.4  +166.3  +223.5 | **<0.0001**  <0.0001  <0.0001  <0.0001 | **27.3**  24.8  30.0  30.7 | **+148.5**  +110.1  +168.7  +196.8 | **<0.0001**  0.0082  0.0044  0.0015 | **43.2**  43.5  40.6  42.8 | **+133.7**  +112.9  +109.2  +197.0 | **<0.0001**  <0.0001  <0.0001  <0.0001 |

**Supplementary Table 10**. Mortality deconvolution of aging *daf-16;* *daf-2* mutant populations with overexpression of individual *daf-16* isoforms from their own promoters.

Worms were maintained at 15˚C and transferred to 20˚C at L4, no FUDR. [C], combined data from all trials, [n], trial number.

| **Genotype** | **Number of deaths/ censored** | **%P** | **All death** | | | **P death** | | | **p death** | | |
| --- | --- | --- | --- | --- | --- | --- | --- | --- | --- | --- | --- |
|  |  |  | **Mean lifespan (days)** | **% change vs. control** | ***p* vs. control (log rank)** | **Mean lifespan (days)** | **% change vs. control** | ***p* vs. control**  **(log rank)** | **Mean lifespan (days)** | **% change vs. control** | ***p* vs. control**  **(log rank)** |
| Wild type (non-roller segregant) (control) | **[C] 154/20**  [1] 98/16  [2] 56/4 | **30.5**  30.6  30.4 | **20.2**  20.1  20.3 |  |  | **13.2**  12.4  14.6 |  |  | **23.2**  23.5  22.8 |  |  |
| *wuEx304 Pmyo-2::daf-16a rol-6(su1006)* | **[C] 123/19**  [1] 73/10  [2] 50/9 | **7.3**  6.8  8.0 | **25.1**  25.1  25.2 | **+24.6**  +24.8  +24.2 | **<0.0001**  <0.0001  <0.0001 | **14.4**  13.6  15.5 | **+9.3**  +9.7  +5.8 | **0.4111**  0.4562  0.8327 | **26.0**  25.9  26.1 | **+11.9**  +10.4  +14.4 | **<0.0001**  0.0025  0.0008 |
| Wild type (non-roller segregant) (control) | **[C] 139/12**  [1] 85/5  [2] 54/7 | **30.9**  31.8  29.6 | **20.4**  20.7  20.1 |  |  | **13.9**  13.6  14.5 |  |  | **23.4**  24.0  22.4 |  |  |
| *wuEx305 Pmyo-2::daf-16f rol-6(su1006)* | **[C] 135/17**  [1] 80/12  [2] 55/5 | **10.4**  12.5  7.3 | **22.1**  21.9  22.4 | **+8.1**  +5.9  +11.7 | **0.1083**  0.5220  0.0490 | **12.9**  12.2  14.8 | **-7.2**  -10.2  +1.7 | **0.3178**  0.3127  0.9682 | **23.2**  23.3  23.0 | **-0.8**  -2.8  +2.5 | **0.6924**  0.5037  0.6963 |

**Supplementary Table 11.** Mortality deconvolution of aging strains with over-expression of individual *daf-16* isoforms in the pharynx.

Trials were performed at 20˚C, no FUDR. [C], combined data from all trials, [n], trial number. Non-roller control were selected from the same plate as roller worms carrying the extrachromosomal transgene array.

| **Genotype** | **Number of deaths/ censored** | **%P** | **All death** | | | **P death** | | | **p death** | | |
| --- | --- | --- | --- | --- | --- | --- | --- | --- | --- | --- | --- |
|  |  |  | **Mean lifespan (days)** | **% change vs. control** | ***p* vs. control (log rank)** | **Mean lifespan (days)** | **% change vs. control** | ***p* vs. control**  **(log rank)** | **Mean lifespan (days)** | **% change vs. control** | ***p* vs. control**  **(log rank)** |
| *daf-16(mgDf50); daf-2(m577)*  (control) | **[C] 102/20**  [1] 51/9  [2] 51/11 | **44.1**  41.2  47.1 | **15.9**  17.1  14.6 |  |  | **10.1**  10.7  9.6 |  |  | **20.4**  21.6  19.1 |  |  |
| *daf-16; daf-2(m577); wuEx304 Pmyo-2::daf-16a* | **[C] 91/19**  [1] 46/12  [2] 45/7 | **6.6**  6.5  6.6 | **22.4**  23.9  20.9 | **+41.1**  +39.8  +42.5 | **<0.0001**  <0.0001  <0.0001 | **10.7**  11.3  10.0 | **+5.7**  +6.3  +4.3 | **0.7808**  0.9372  0.7381 | **23.2**  24.7  21.6 | **+13.7**  +14.7  +13.0 | **<0.0001**  <0.0001  0.0045 |
| *daf-16; daf-2(m577); wuEx305 Pmyo-2::daf-16f* | **[C] 93/18**  [1] 53/7  [2] 40/11 | **11.8**  11.3  12.5 | **18.5**  18.4  18.8 | **+16.9**  +7.6  +28.2 | **0.0281**  0.9028  0.0022 | **13.8**  15.7  11.6 | **+37.0**  +46.9  +21.0 | **0.0009**  0.0083  0.0214 | **19.2**  18.7  19.8 | **-6.1**  -13.2  +3.4 | **0.3180**  0.0301  0.4600 |
| *daf-16; daf-2(e1370)*  (control) | **[C] 97/24**  [1] 46/14  [2] 51/10 | **50.5**  52.2  49.1 | **15.1**  15.3  14.9 |  |  | **10.4**  10.8  10.1 |  |  | **19.9**  20.3  19.5 |  |  |
| *daf-16; daf-2(e1370); wuEx304 Pmyo-2::daf-16a* | **[C] 96/18**  [1] 52/8  [2] 44/10 | **6.3**  5.8  6.8 | **24.1**  25.4  22.6 | **+59.4**  +65.3  +51.6 | **<0.0001**  <0.0001  <0.0001 | **16.8**  16.7  17.0 | **+61.1**  +53.8  +68.7 | **0.0022**  0.0579  0.0097 | **24.6**  25.9  23.0 | **+23.7**  +27.7  +17.7 | **<0.0001**  <0.0001  0.0016 |
| *daf-16; daf-2(e1370); wuEx305 Pmyo-2::daf-16f* | **[C] 89/21**  [1] 45/11  [2] 44/10 | **5.6**  4.4  6.8 | **26.1**  27.2  24.9 | **+72.5**  +77.2  +67.2 | **<0.0001**  <0.0001  <0.0001 | **10.4**  10.0  10.7 | **-0.5**  -7.7  +5.8 | **0.6152**  0.4541  0.6472 | **27,0**  28.0  26.0 | **+35.8**  +38.1  +32.8 | **<0.0001**  <0.0001  <0.0001 |

**Supplementary Table 12.** Mortality deconvolution of aging *daf-16;* *daf-2* mutant populations with overexpression of individual *daf-16* isoforms in the pharynx.

Trials were performed at 20˚C, with no FUDR. [C], combined data from all trials, [n], trial number.

| **Genotype** | **Number of deaths/ censored** | **%P** | **All death** | | | **P death** | | | **p death** | | |
| --- | --- | --- | --- | --- | --- | --- | --- | --- | --- | --- | --- |
|  |  |  | **Mean lifespan (days)** | **% change vs. control** | ***p* vs. control (log rank)** | **Mean lifespan (days)** | **% change vs. control** | ***p* vs. control**  **(log rank)** | **Mean lifespan (days)** | **% change vs. control** | ***p* vs. control**  **(log rank)** |
| N2  (control) | **[C] 106/21**  [1] 60/7  [2] 46/14 | **49.1**  48.3  50.0 | **11.3**  10.9  11.7 |  |  | **8.3**  8.1  8.6 |  |  | **14.1**  13.5  14.9 |  |  |
| *daf-12(m20)* | **[C] 109/22**  [1] 60/12  [2] 49/10 | **71.6**  70.0  73.5 | **9.4**  9.6  9.1 | **-16.8**  -12.4  -22.1 | **0.0003**  0.0833  0.0027 | **7.7**  7.9  7.6 | **-7.1**  -3.0  -11.8 | **0.0142**  0.2857  0.0560 | **13.5**  13.5  13.5 | **-4.2**  -0.1  -9.2 | **0.3156**  0.8680  0.0951 |
| *daf-2(e1368)*  (control) | **[C] 105/23**  [1] 58/7  [2] 47/16 | **30.5**  32.8  27.7 | **23.6**  21.4  26.4 |  |  | **12.5**  12.1  13.1 |  |  | **28.5**  25.9  31.4 |  |  |
| *daf-2(e1368); daf-12(m20)* | **[C] 109/21**  [1] 60/6  [2] 49/15 | **40.4**  43.3  36.7 | **22.2**  20.4  24.5 | **-5.8**  -4.7  -7.1 | **0.6785**  0.8490  0.4984 | **10.1**  10.3  9.7 | **-19.3**  -14.2  -26.0 | **0.0033**  0.0816  0.0084 | **30.5**  28.1  33.1 | **+6.9**  +8.2  +5.3 | **0.3540**  0.3069  0.3872 |
| *daf-2(m41)*  (control) | **[C] 89/38**  [1] 42/26  [2] 47/12 | **29.2**  35.7  23.4 | **27.3**  23.0  31.1 |  |  | **12.8**  14.9  9.8 |  |  | **33.3**  27.6  37.6 |  |  |
| *daf-2(m41); daf-12(m20)* | **[C] 92/36**  [1] 49/15  [2] 43/21 | **55.4**  53.1  58.1 | **21.4**  20.8  22.1 | **-21.6**  -9.9  -28.9 | **0.0073**  0.2326  0.0956 | **11.4**  12.3  10.4 | **-10.8**  -17.3  +5.9 | **0.1488**  0.0452  0.4816 | **33.9**  30.3  38.4 | **+1.6**  +9.8  +2.1 | **0.9812**  0.6143  0.5211 |
| *daf-2(e1370)*  (control) | **[C] 61/74**  [1] 35/35  [2] 26/39 | **0.0**  0.0  0.0 | **34.9**  34.0  36.2 |  |  |  |  |  | **34.9**  34.0  36.2 |  |  |
| *daf-2(e1370); daf-12(m20)* | **[C] 104/17**  [1] 60/10  [2] 44/7 | **0.0**  0.0  0.0 | **37.8**  37.1  38.7 | **+8.2**  +9.2  +7.0 | **0.4487**  0.5837  0.5195 |  |  |  | **37.8**  37.1  38.7 | **+8.2**  +9.2  +7.0 | **0.2452**  0.3208  0.4222 |

**Supplementary Table 13.** Mortality deconvolution of aging *daf-2; daf-12(m20)* mutant populations at 25˚C.

Worms were maintained at 15˚C and transferred to 25˚C at L4, no FUDR. [C], combined data from all trials, [n], trial number.

| **Genotype** | **Number of deaths/ censored** | **%P** | **All deaths** | | | **P deaths** | | | **p deaths** | | |
| --- | --- | --- | --- | --- | --- | --- | --- | --- | --- | --- | --- |
|  |  |  | **Mean lifespan (days)** | **% change vs. control** | ***p* vs. control (log rank)** | **Mean lifespan (days)** | **% change vs. control** | ***p* vs. control**  **(log rank)** | **Mean lifespan (days)** | **% change vs. control** | ***p* vs. control**  **(log rank)** |
| N2 (control) | **[C] 137/17**  [1] 46/3  [2] 43/2  [3] 48/12 | **35.8**  39.1  39.5  29.2 | **17.8**  18.7  16.2  18.4 |  |  | **11.5**  12.4  9.8  12.3 |  |  | **21.3**  22.8  20.3  20.9 |  |  |
| *daf-12(rh61rh411)* | **[C] 159/9**  [1] 56/2  [2] 52/1  [3] 51/6 | **57.9**  41.9  57.7  58.8 | **13.3**  13.4  13.2  13.1 | **-25.6**  -28.2  -18.2  -28.6 | **<0.0001**  <0.0001  0.0033  <0.0001 | **10.6**  10.9  10.9  10.0 | **-7.7**  -12.6  +11.3  -18.6 | **0.0316**  0.0093  0.1737  0.0142 | **16.9**  16.9  16.2  17.6 | **-20.8**  -25.8  -20.3  -15.7 | **<0.0001**  0.0001  0.0031  0.0100 |

Supplementary Table 14. Mortality deconvolution of aging N2 and *daf-12* null mutant populations at 20˚C.

Trials were performed at 20˚C, with no FUDR. [C], combined data from all trials, [n], trial number.
